# Micronutrient Deficiency and Treatment Adherence in a Randomized Controlled Trial of Micronutrient Supplementation in ART-Naïve Persons with HIV

**DOI:** 10.1371/journal.pone.0085607

**Published:** 2014-01-21

**Authors:** Louise Balfour, Johanna N. Spaans, Dean Fergusson, Harold Huff, Edward J. Mills, Charles J. la Porte, Sharon Walmsley, Neera Singhal, Ron Rosenes, Nancy Tremblay, M. John Gill, Hugues Loemba, Brian Conway, Anita Rachlis, Edward Ralph, Mona Loutfy, Ranjeeta Mallick, Rika Moorhouse, D. William Cameron

**Affiliations:** 1 CIHR Canadian HIV Trials Network (CTN), Vancouver, British Columbia, Canada; 2 Division of Infectious Diseases, Department of Medicine, University of Ottawa at the Ottawa Hospital, Ottawa, Ontario, Canada; 3 Clinical Epidemiology Program, University of Ottawa at The Ottawa Hospital Research Institute (OHRI), Ottawa, Ontario, Canada; 4 Canadian College of Naturopathic Medicine, Toronto, Ontario, Canada; 5 Faculty of Health Sciences, University of Ottawa, Ottawa, Ontario, Canada; 6 Division of Infectious Diseases, Department of Medicine, University of Toronto, Toronto, Ontario, Canada; 7 Department of Microbiology, Immunology and Infectious Diseases, University of Calgary, Calgary, Alberta, Canada; 8 Department of Medicine, The University of Ottawa, Ottawa, Ontario, Canada; 9 Department of Anesthesiology, Pharmacology and Therapeutics, University of British Columbia, Vancouver, British Columbia, Canada; 10 Division of Infectious Diseases, Department of Medicine, University of Western Ontario, London, Ontario, Canada; 11 Ottawa Methods Centre, Clinical Epidemiology Program, Ottawa Hospital Research Institute, Ottawa, Ontario, Canada; Institute of Infectious Diseases and Molecular Medicine, South Africa

## Abstract

**Introduction:**

The MAINTAIN study is an on-going RCT comparing high-dose micronutrient and anti-oxidant supplementation versus recommended daily allowance (RDA) vitamins in slowing HIV immune deficiency progression in ART-naïve people with HIV infection.

**Objective:**

We planned analysis of the first 127 participants to determine the baseline prevalence of serum micronutrient deficiencies and correlates, as well as tolerance and adherence to study interventions.

**Methods:**

Participants receive eight capsules twice daily of 1) high-dose or 2) RDA supplements for two years and are followed-up quarterly for measures of immune deficiency progression, safety and tolerability. Regression analysis was used to identify correlates of micronutrient levels at baseline. Adherence was measured by residual pill count, self-report using the General Treatment Scale (GTS) and short-term recall HIV Adherence Treatment Scale (HATS).

**Results:**

Prior micronutrient supplementation (within 30 days) was 27% at screening and 10% of study population, and was not correlated with baseline micronutrient levels. Low levels were frequent for carotene (24%<1 nmol/L), vitamin D (24%<40 nmol/L) and serum folate (20%<15 nmol/L). The proportion with B_12_ deficiency (<133 pmol/L) was 2.4%. Lower baseline levels of B_12_ correlated lower baseline CD4 count (r = 0.21, p = 0.02) with a 21 pmol/L reduction in B_12_ per 100 cells/µL CD4. Vitamin D levels were higher in men (p<0.001). After a median follow-up of 1.63 years, there were 19 (15%) early withdrawals from the study treatment. Mean treatment adherence using pill count was 88%. Subjective adherence by the GTS was 81% and was moderately but significantly correlated with pill count (r = 0.29, p<0.001). Adherence based on short-term recall (HATS) was >80% in 75% of participants.

**Conclusion:**

Micronutrient levels in asymptomatic HIV+ persons are in keeping with population norms, but micronutrient deficiencies are frequent. Adherence levels are high, and will permit a valid evaluation of treatment effects.

**Trial Registration:**

ClinicalTrials.gov NCT00798772

## Introduction

Many HIV/AIDS patients choose to use complementary therapies as a manner of dealing with health maintenance, adverse events, or as a primary treatment for HIV infection. The evidence for complementary therapies in HIV/AIDS treatment is sparse. We are currently conducting a randomized trial to assess the role of high-dose micronutrient supplementation with vitamins, minerals and antioxidants compared with a control group receiving recommended daily allowance (RDA) of a micronutrient supplementation of vitamins and minerals. The MAINTAIN trial (clinicaltrials.gov NCT00798772) aims to determine if micronutrient supplementation can delay HIV immune deficiency progression and delay the need to start anti-retroviral treatment (ART) among HIV-infected ART- naïve study volunteers.

There are several key elements of the trial that require consideration if our trial is to have good external and internal validity. Chief among these is adherence to the prescribed interventions. Low adherence to long-term medicinal treatments is a leading cause of suboptimal therapeutic benefit in the medical management of chronic diseases, especially in diseases with minimal symptoms [1]. In persons living with HIV/AIDS, low treatment adherence to ART results in incomplete viral suppression and may increase the risk of drug resistant HIV infection, progressive immune deficiency and clinical treatment failure [2], [3]. Adherence is driven by several factors, including tolerance, toxicities, frequency of dosing and pill counts, as well as motivation, and perceptions of benefit or harm by the physician and the patient [4]–[7]. Little is known about adherence to complementary therapies, although these may be subject to the same determinants of treatment adherence as ART. These adherence factors also affect trial design [8]. Measuring adherence to prescribed trial medication is therefore important for assessing the trial intervention’s broad practicability and to assess attrition bias within the study.

A further consideration for internal validity is the adequate assessment of micronutrient deficiency. It has long been recognized that people with HIV suffer from micronutrient deficiencies as a result of dietary micronutrient inadequacy despite macronutrient adequacy, of HIV-associated enteric malabsorption, and of consumptive depletion due to increased metabolic demand and inflammatory oxidative stress [9], [10]. Supplementation has therefore been proposed to correct micronutrient deficiencies in HIV [11], [12], and supra-physiologic dosing of vitamins and antioxidants may address the inflammation and oxidative stress, which are all components of HIV immunopathogenesis. A review of supplementation trials [13] suggests that multiple micronutrient supplements may confer multiple clinical benefits over single supplements, particularly in pregnant women and their offspring. In the largest randomized trial, multiple micronutrient supplements to date, high-dose micronutrient supplements during the second and third trimester and lactation in Tanzanian women delayed disease progression or AIDS-related mortality [HR = 0.71, 95% CI = 0.51–0.90] and reduced the risk of low birth weight infants compared to placebo [14]. While these reports have received some support by others, it has been suggested that the benefit of micronutrient supplementation may be restricted to those with lower CD4 counts [15] and those with lower micronutrient levels [16]. It is therefore critical to assess micronutrient levels in the study population at baseline to draw valid inference and properly characterize the potential benefits of micronutrient supplementation.

In order to examine these key aspects of trial validity, we pre-planned a mid-study sub-analysis. We aimed to evaluate the prevalence of prior micronutrient supplementation, the prevalence of micronutrient deficiencies as measured by serum levels at randomization, and the correlation of low micronutrient levels with other baseline characteristics, as well as safety, tolerance, and treatment adherence to this burdensome 16 micronutrient capsules/day regimen over time.

## Methods

### Study Design

Our study methods and design have previously been published [17]. Briefly, the MAINTAIN study is a two-arm RCT to evaluate the effect of high-dose micronutrient supplements on measures of HIV disease progression used to guide ART initiation. Participants are enrolled in the study during routine clinic visits. Research staff conduct a preliminary screening, explain the trial to the potential participant, carry out consent procedures, and screen for eligibility to participate in the study. After screening and consent, the study nurse obtains an identification number for the participant from a study randomization website. The randomization process consists of a computer-generated random listing of the treatment allocations. A permuted blocked randomization method is used to allocate participants to a study group, in variable blocks of two and four, stratified by centre and CD4 T lymphocyte count balanced above and below 500 cells/mm^3^. Study participants receive either supplement with a high-dose micronutrient, mineral and antioxidant preparation (K-PAX Ultra®) or an identically appearing 100% RDA preparation of multivitamins and minerals. Participants are required to take one packet of eight capsules with water, in mid-meal with water twice daily (16 capsules per day) for two years. Participants, physicians, nurses, investigators, medication dispensers and research staff are blinded to the randomization schemes and treatments administered. To maintain blinding, an independent biostatistician is assigned to prepare randomization schemes and perform interim analyses. At the coordinating centre, only a designated research pharmacist is aware of the treatment allocation of individual participants.

The primary outcome is a composite measure of time from randomization to confirmed CD4 T lymphocyte count <350 cells/µL or emergence of documented CDC-defined AIDS-defining illness or start of ART. With a planned sample size of 218, the study will have 80% power to detect a decrease in the median event rate at 2 years from 50% to 32% at the 0.05 level of significance, assuming a protocol or follow-up non-compliance rate of up to 20%. This study is being conducted in accordance with the ethical standards on human experimentation of the Declaration of Helsinki. It has been approved by the local research ethics board at each of the participating CIHR Canadian HIV Trials Network (CTN) research centres across Canada including: Ottawa Health Science Network Research Ethics Board, Capital Health Research Ethics Board, University of British Columbia Office of Research Services, Institutional Review Board Services, UBC-Providence Health Care Research Ethics Board, Queen’s University Health Sciences and Affiliated Teaching Hospitals Research Ethics Board, comite d’éthique de la recherche du CHUQ-CHUL, Biomedical A Research Ethics Board of the MUHC, Sunnybrook Health Sciences Centre Research Ethics Board, Conjoint Health Research Ethics Board, University of Western Ontario Health Sciences Research Ethics Board, Women’s College Hospital Research Ethics Board, Comité d’éthique de la recherche du CHUM, University of Manitoba Bannatyne Campus Research Ethics Board, Hamilton Health Sciences/Faculty of Health Sciences Research Ethics Board, Hamilton Integrated Research Ethics Board, Windsor Regional Hospital Research Ethics Board, Sudbury Regional Hospital Research Ethics Committee, and Health Research Ethics Authority. Written informed consent was obtained from all study participants.

### Study Participants

Volunteers are eligible to participate if they are asymptomatic HIV-infected individuals with a CD4 T cell count between 375–750 cells/µL at screening evaluation. Study participants are not eligible if they had ever been on ART (excluding less than seven days duration, and perinatal transmission prophylaxis). Exclusion criteria include HIV-2 infection alone, pregnancy or not willing to practice barrier method of birth control, on treatment for ongoing opportunistic infection and taking micronutrient or natural health product supplements that may overlap study medication components within thirty days of randomization. For this investigation on baseline micronutrient deficiency and on treatment adherence, analysis is limited to the first 127 randomized participants in whom adequate baseline and follow-up treatment adherence data was available at the time of analysis.

### Evaluations

Following the baseline assessment and randomization, study participants are evaluated every twelve weeks for measures of disease progression in parallel with standard clinical care. At each scheduled clinic visit, study participants have a physical exam, including anthropometrics and a review of adverse events, serum chemistries and micronutrient levels, plasma HIV-RNA levels, and peripheral blood lymphocyte immunophenotype subset measures. At each quarterly study visit participants return their study treatment medication containers with any unused study medication, and received a 90-day supply of the assigned study treatment. Treatment adherence is calculated using the residual pill count at the return visit and the interval in days from the prior dispensing visit. Self-reported treatment adherence is evaluated using the self-administered HIV Treatment Adherence Scale [18], which measures treatment adherence based on the number of missed doses in the previous week and the General Treatment Scale [19], a validated 5-item Likert questionnaire that measures treatment adherence based on the degree of agreement with questions such as “I follow my doctor’s suggestions exactly”, with scores ranging from 0 (none of the time) to 5 (all of the time) for each scale item. Overall GTS scores are calculated by summing across scale items, and are expressed as a summary percentage adherence estimate.

### Micronutrient Analysis

Blood samples are drawn for serum chemistries and micronutrient analysis. Samples are processed locally in licensed diagnostic laboratories that are subject to regulatory certification of quality assurance. At The Ottawa Hospital (national study centre), carotene is analysed using spectrophotometry (Beckman Coulter DU 702 spectrophotometer), B_12_ is measured with a two-site competitive binding immune-enzymatic assay with chemiluminescence (Beckman Coulter Unicel® Dxl 800) and 25-hydroxyvitamin D analysis uses a chemiluminescence immunoassay (IDS-iSYS). Serum folate is assessed using a two-step competitive binding receptor assay with chemiluminescence (Beckman Coulter Unicel ® Dxl 800). At two centres, erythrocyte folate is measured using a competitive binding assay with chemiluminescence (ADVIA Centaur® Folate Assay or Beckman Coulter Unicel® Dxl 800).

### Statistical Analysis

**Figure 1:**
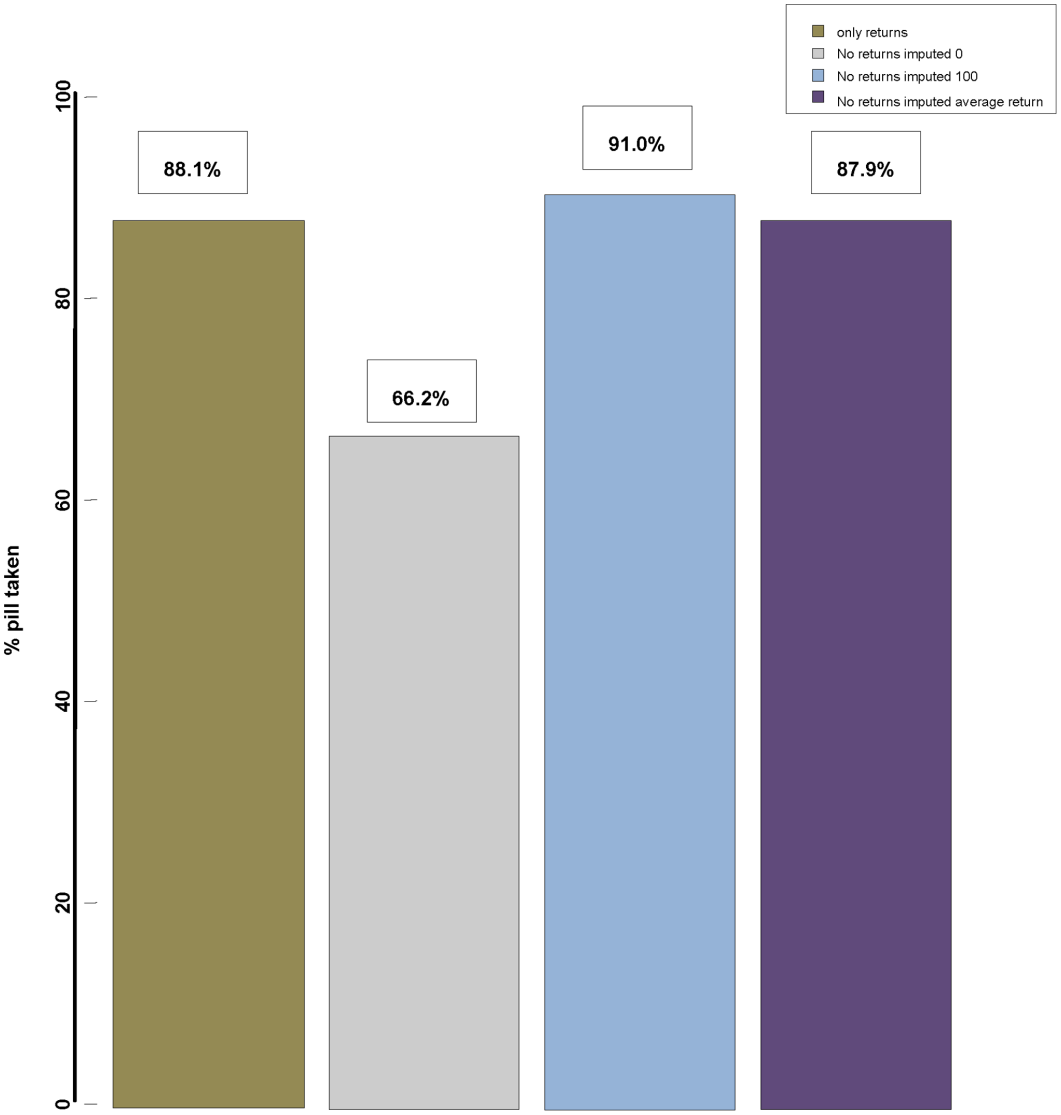
Average treatment adherence by returned capsule count, and with multiple imputation for non-returns. Adherence to study medication over time was assessed by counting capsule packets returned at weeks 12, 24, 36, 48, 60, 72 and 84. Treatment adherence was calculated and compared using different methods. When only those who attended visits and returned capsule packets were included in analysis, average adherence was 88%. Intention-to-treat analysis, with 26% imputation for missing returns, yielded three estimates: 66% (0% imputed for missing values), 91% (100% imputed for missing values), and 88% (average adherence of returns imputed for missing values).

For the purpose of the current investigation on treatment adherence and micronutrient deficiency, allocation-blinded data on baseline micronutrient levels, residual pill count, patient self-reported GTS and HATS adherence scales were limited to the first 127 randomized study participants. To preserve blinding, serial serum micronutrient level measures were not evaluated. Treatment adherence was calculated as a percentage of actual versus the expected capsule consumption, inferred from unreturned packets. Overall mean treatment adherence was determined by averaging across time points up week 84. Treatment adherence at each time point was also determined and presented using descriptive statistics, to evaluate the impact of the duration of therapy on treatment adherence. For missing values, mean adherence for those who attended their scheduled visits and returned capsule packets was imputed. Two additional imputation methods, and documented pill returns only, are included in figure 1 for comparison of mean adherence estimates. Self-reported treatment adherence was determined using the HATS and GTS. The correlation between pill count and self-reported treatment adherence on the GTS was calculated using the Pearson correlation coefficient for overall treatment adherence. Finally, descriptive statistics were calculated for serum micronutrient levels of carotene, 25-OH vitamin D, vitamin B_12_ and folate. Low serum levels of micronutrients were defined at thresholds: carotene <1 µmol/L, vitamin D<75 nmol/L (insufficient), <40 nmol/L (deficient) or <20 nmol/L (significantly deficient) of 25-OH vitamin D, and vitamin B_12_<133 pmol/L. Folate thresholds were either <15 nmol/L) or <450 nmol/L depending on the assay used. To capture results from both assays, folate deficiency is also reported categorically.

A completed CONSORT checklist is available as supporting information; see Checklist S2) CONSORT Checklist.

## Results

**Figure 2:**
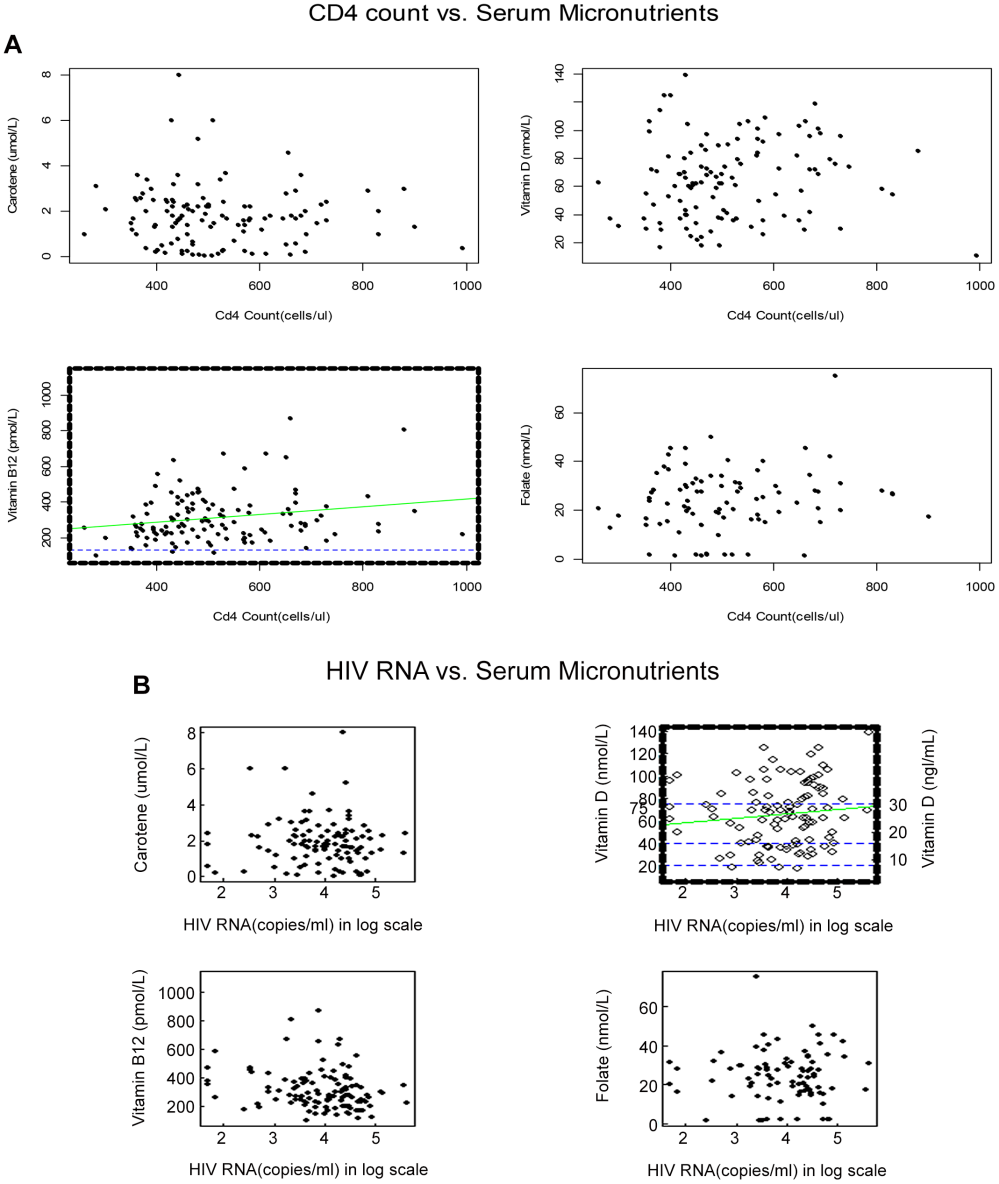
**A. CD4 count (cells/mL) vs. serum micronutrients.** The B_12_ threshold of 133 pmol/L demonstrates that 3 (2.4%) of baseline measures are below the lower limit of the normal range. The slope (21 pmol/L per 100 cells/µL) indicates the relationship between CD4 lymphopenia and serum B_12_ levels, which are largely within the normal range for B_12_. **B. Plasma virus load (log10 copies/mL) vs. serum micronutrients.** The 25-OH D thresholds indicate that 67%, 24% and 3.5% of baseline measures indicate insufficiency (<75 nmol/L), deficiency (<40 nmol/L) and severe deficiency (<20 nmol/L), respectively. Higher baseline levels of vitamin D were more common in men (p<0.001) and correlated higher baseline plasma HIV-RNA level (p = 0.02). All other demographic and baseline clinical factors were not significantly associated with baseline micronutrient levels.

The trial began enrolling in March 2009. As of June 2013, 171 participants had been enrolled to the trial. Our blinded analysis of the first 127 study participants was based on a median of 1.63 years [IQR (0.8,1.85)] follow up. The mean age at baseline was 38.1 [SD 8.9] years. Micronutrient or vitamin D supplementation was reported at screening in 27% and 10% of the study population, respectively. The demographic and clinical characteristics of patients screened for study eligibility and the subset enrolled to the trial are presented in Tables 1 and 2, with baseline characteristics for enrolled participants in the trial in Table 3 and 4. Baseline micronutrient levels and deficiency thresholds for study participants are presented in Table 5. Low serum micronutrient levels were common among the study participants at baseline, with 24% having serum carotene levels below the population norm (<1.0 µmol/L). Vitamin D levels were insufficient (<75 nmol/L) in 67%, deficient (<40 nmol/L) in 24%, and severely deficient (<20 nmol/L) in 3.5%. When all evaluated categorically using both serum and erythrocyte folate measures, 23% had folate levels below threshold. Vitamin B_12_ levels were in the deficient range (<133 pmol/L) for three (2.4%) study participants. Lower baseline levels of B_12_ correlated lower baseline CD4 count (r = 0.2, p = 0.007) in multiple linear regression adjusted for sex and BMI, as well as in unadjusted analysis (r = 0.21, p = 0.02, Figure 2a). Higher baseline levels of vitamin D correlated higher baseline plasma HIV-RNA level in unadjusted analysis (r = 0.22, p = 0.02, Figure 2b). In multiple linear regression adjusted for sex and BMI, correlation was high but not significant (r = 0.97, p = 0.5). Higher baseline levels of vitamin D were more common in men (p<0.001). All other demographic and baseline clinical factors were not significantly associated with baseline micronutrient levels. Baseline micronutrient levels or deficiency did not correlate with prior supplementation.

**Table 1:** Demographic characteristics for randomized and non-randomized patients at screening.

Characteristic	Category	N (%) randomized	N (%) screened, not randomized
**Sex**	Male	105 (82.7)	30 (79.0)
	Female	22 (17.3)	8 (21.1)
**Ethnicity**	Asian	3 (2.4)	0 (0.0)
	Black	23 (18.1)	10 (26.3)
	Caucasian	85 (66.9)	21 (55.3)
	Native	5 (3.9)	4 (10.5)
	Other	11 (8.7)	4 (10.5)

**Table 2:** Demographic and clinical characteristics for randomized and non-randomized patients at screening.

	Randomized			Screened, not randomized		
Characteristic	N	Mean (SD)	Median (IQR)	N	Mean (SD)	Median (IQR)
Age (years)	127	38.1 (8.9)	38.1 (31.3, 45.9)	38	40.4 (10.3)	40.9 (35.2, 45.6)
BMI	120	25.7(5.2)	25.3 (22.8, 28.6)	33	25.8 (4.8)	26.5 (22.6, 28.6)
HIV-1 RNA (copies/mL)	127	23 428 (41 525)	9100 (3060, 26 951)	37	35262 (77 312)	8464 (4168, 23 292)
CD4 count (cells/µL)	127	525 (104)	509 (440, 600)	36	454 (177)	384 (326, 510)
CD8 count (cells/µL)	127	970 (478)	823 (641, 1 175)	36	869 (367)	830 (625, 1085)
CD4:CD8 (ratio)	124	0.64 (0.32)	0.6 (0.42, 0.8)	35	0.6 (0.29)	0.52 (0.42, 0.89)
Time since diagnosis(years)	106	2.4 (3.2)	1.2 (0.4, 3.1)	31	2.3 (3.1)	1.3 (0.3, 2.8)

**Table 3:** Demographic characteristics for randomized participants at baseline.

Characteristic	Category	N (%)
**Sex**	Male	105 (82.7)
	Female	22 (17.3)
**Ethnicity**	Asian	3 (2.4)
	Black	23 (18.1)
	Caucasian	85 (66.9)
	Native	5 (3.9)
	Other	11 (8.7)

**Table 4:** Demographic and clinical characteristics for randomized participants at baseline.

Characteristic	N	Mean (SD)	Median (IQR)
Age (years)	127	38.1 (8.9)	38.1 (31.3, 45.9)
BMI	126	25.8 (4.3)	25.4 (22.9, 28.5)
HIV-1 RNA (copies/ml)	117	29352 (64 314)	11 269 (2849, 28 660)
CD4 count (cells/µL)	126	515 (133)	486 (428, 580)
CD8 count (cells/µL)	126	953 (506)	838 (620, 1 156)
CD4:CD8 (ratio)	123	0.65 (0.33)	0.61 (0.42, 0.8)

**Table 5:** Baseline Micronutrient Levels.

Serum Micronutrients	N	Mean (SD)	Median (IQR)	Micronutrient Deficiency
Carotene (µmol/L)*	117	1.8 (1.3)	1.7 (1, 2.4)	24% <1
Vitamin D (nmol/L)	115	64.9 (27.7)	66 (40, 84)	67% <75 24% <40 3.5% <20
Vitamin B_12_ (pmol/L)*	123	319 (153.7)	278 (222, 384)	2.4% <133
Folate (nmol/L)^∧^ *	88+	23.8 (13.0)	23.9 (16.3, 31)	20% <15+

* No significant differences based on sex were observed in micronutrient levels.

^∧^ Reported folate levels exclude baseline values for erythrocyte folate (n = 11).

+ When evaluated categorically using both serum and erythrocyte folate measures (n = 99), 23% had folate levels below threshold.

### Residual Pill Count

**Figure 3:**
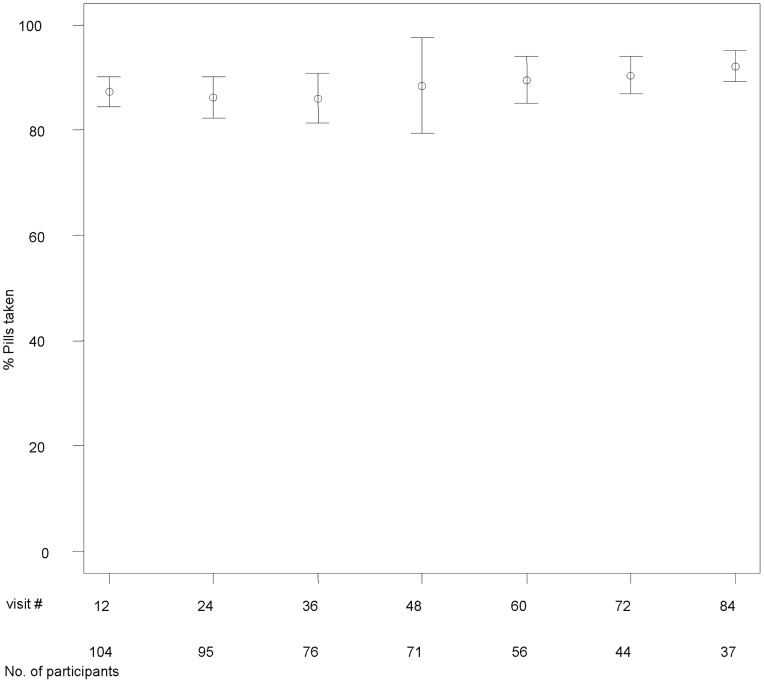
Treatment adherence by residual pill count over time. The data points and bars indicate mean percentage and standard deviation of the expected unreturned medication capsules on the Y-axis. The X-axis indicates the study follow-up visit week. The number of subjects included in analysis is included under the X-axis. Imputation of average adherence was used for missing values.

Treatment adherence was calculated at each three-month interval. As shown in Figure 3, mean treatment adherence was greater than 86% at each time point up to week 84 and demonstrated little change over time. Overall mean treatment adherence across time points was 88%. Among study participants with a minimum of 12 months of follow-up, mean treatment adherence was 89%. Twenty-six percent of pill count adherence data were missing and was evaluated with various imputations (figure 1).

### Self-reported Adherence

Based on short-term recall using the HATS, self-reported treatment adherence was good (>80%) in 75% of the study population. In this sample, the GTS had a Chronbach’s Alpha score of 0.76, indicating adequate internal reliability [20] and the GTS’s inter-item correlation was.41 which is good [21]. Self-reported treatment adherence using the GTS was lower than objective pill count measures of treatment adherence (81% vs 88%). The degree of correlation between these subjective and objective measures of treatment adherence over the 84-week interval was moderate but highly significant (r = 0.29, p<0.001). Qualitative responses on adherence indicate that the majority of missed doses were due to participants forgetting to take protocol medication. Busy lifestyle, treatment intolerance (e.g. hard to swallow, nausea), and unrelated health complications were also reported as reasons for missed dosages.

### Safety, Protocol Compliance and Follow-up

There have been a total of 21 serious adverse events reported, related to 15 individual participants, none were deemed to be treatment related. These serious adverse events comprise appendicitis, community-acquired pneumonia, cholecystitis, perianal abscess, perianal tumefaction, attempted suicide, unexplained fever, lymphadenopathy, benzodiazepine overdose, depression, septic arthritis, suppurative cholecystitis, intra-abdominal abscess, and psychosis. In addition, there have been 19 (15%) early withdrawals from the study treatment among the first 127 people randomized. Reasons given for study withdrawal include pill burden (n = 4), intolerance (n = 4), lost interest/lost to follow up (n = 4), competing trial (n = 3), side effects (n = 2), non-compliance (n = 2), schedule conflict (n = 1), and termination by sponsor (n = 1). ‘Intolerance’ includes nausea, difficulty swallowing pills, and pill taste. Study participants continue in follow-up regardless, unless there is withdrawal of consent.

## Discussion

We found that baseline micronutrient deficiencies were common in the study population. While the mean serum levels of vitamin D and vitamin B_12_ observed were comparable to the values seen in the general adult population aged 20–59 [22], a high proportion of individuals within the study population were deficient on one or more serum micronutrient measures. Of note, prior micronutrient (within 30 days of screening) was not correlated with baseline micronutrient levels, suggesting that formulations with standard micronutrient RDA supplementation may be inadequate among those with HIV infection. The weak but significant correlation of levels of serum vitamin B_12_ with CD4 count observed may suggest either that low B_12_ levels may predict CD4 decline, or that B_12_ and CD4 count decline in concert. The correlation of higher serum vitamin D with greater plasma viremia levels is interesting. Higher vitamin D levels also correlated with gender. Confounders, such as race or ethnicity, season and sunlight exposure, artificial tanning, and high dose supplementation may be considered in analysis of the whole study population.

Based on the blinded preliminary results presented here, adherence to the assigned micronutrient treatments is acceptably high (>80%) on both objective and subjective measures of treatment adherence, despite the high pill burden. Rates of treatment adherence improved slightly with time and rates at twelve months were still acceptable. Study participant withdrawal and adverse event rates in this trial are low and within projections, likely owing to the good safety profile of the treatments, clinically driven on-study follow-up schedule and a self-selected, motivated study population.

There are both strengths and weaknesses to interpreting our analyses. We performed this analysis to ensure that the un-blinded findings of our study will be robust and can be interpreted with confidence about the study intervention, patient status and adherence, and involved all patients enrolled according to our pre-planned analysis. There is no gold standard measure of adherence Treatment adherence was assessed indirectly and is subject to biases. Counting returned capsules may overestimate adherence since this method assumes that all missing capsules have been ingested. Imputing average adherence for missing data, including missed visits, may also overestimate adherence since missed appointments are often a predictor of low adherence [23]–[25]. Social desirability may influence participants to exaggerate compliance on the qualitative self-report measures of adherence. In the un-blinded analysis of final study results, serum level measures of will be used. Although, this includes its own biases.

Study participants are likely a representative sample of the eligible population, given the comparability of baseline demographic and clinical characteristics between people screened for eligibility and enrolled to the trial. A weakness of this analysis though is that it is based on a subset of the target study population, such that some findings may change with study completion and that some associations may be spurious despite significance due to chance, through multiple comparisons or due to confounding of other associations.

Our results are comparable to the mean levels of treatment adherence in the Tanzanian study, described previously, during pregnancy (91%) and at three months post-partum (88%) [14]. While not as high as the treatment adherence reported by Allard and colleagues following short-course treatment with vitamin E and C in ART-treated individuals [26], the lower pill burden (two capsules and two tablets daily) and shorter duration of treatment (three months) may have accounted for the higher treatment adherence in that trial. Notably, the mean treatment adherence at three months based on pill count in the MAINTAIN study was 87%, despite the sixteen capsules consumed daily in our study.

Our long-term results are much better than those reported by Semba, who evaluated micronutrient supplementation in hepatitis C infected individuals, 30% of whom were co-infected with HIV [27]. In the Semba study, although the overall percentage of people achieving >80% treatment adherence was acceptable in both treatment arms (74% and 77%), the reported drop-out rate during the one year of treatment was 27.7% [27]. Given that the study was limited to women with a history of injection drug use, however, the high drop-out rates are not unexpected or representative of a wider population.

Self-reported treatment adherence in our study was also high and showed a modest degree of correlation with our objective measure of treatment adherence. Of interest, the levels of treatment adherence reported herein are much greater than has sometimes been reported for ART [28]. The high level of compliance in this study though is not surprising. Participants who are willing to volunteer for a study with burdensome treatment regimens are a motivated group. In addition, ‘vitamins’ are considered beneficial by the general population and supplementation in the HIV community is widespread and often patient-initiated. The potential for treatment acceptability in a wider population though must be considered; despite low participant attrition in this self-selected population, pill burden and intolerance were cited as reasons for trial withdrawal. The role of micronutrient supplementation in stabilizing or delaying immune decline in persons not on ART can only be evaluated within a controlled clinical trial. A pharmaceutical grade formulation of the high-dose micronutrients was chosen for the experimental intervention in this clinical trial because of its bioavailability and stability, which has been problematic in other settings [16], [29]. While demanding of participants, the 16-capsule daily regimen allows for a more definitive trial given pharmaceutical stability and adherence. If high-dose micronutrient supplementation demonstrates significantly better outcomes compared to standard dose micronutrients, options for less demanding pill burden should be considered.

We are encouraged by the preliminary results of treatment adherence in the MAINTAIN study. The good treatment adherence observed is consistent with the perceived health benefit of micronutrient supplementation, widely held among the general public despite lack of good evidence. The high pill burden of the study treatment regimens and duration of therapy appear to be acceptable to asymptomatic untreated HIV-infected individuals, in part owing to their good safety profile and likely due to the motivation of patient volunteers.

Although ART recommendations accommodate early initiation above 350 CD4+ cells/µl, in practice there are deterrents to treatment, including fears of toxicities, cost and the long-term requirement for continuous ART. With either a positive or negative outcome, this trial will offer valid and improved knowledge of the real value of a commonly used and relatively uninformed health maintenance practice for living with HIV.

## Supporting Information

Checklist S1 The completed CONSORT checklist indicates sections of the publication in which checklist items can be found.
(PDF)

File S1 **The MAINTAIN Study Group.** Full list of investigators, study coordinators, and data management personnel participating in the MAINTAIN study.
(DOCX)

Protocol S1 (PDF)

## References

[1] Dunbar-Jacob J, Erlen JA, Schlenk EA, Ryan CM, Sereika SM, et al. (2000) Adherence in chronic disease. Annu Rev Nurs Res 18: 48–90. Available: http://www.ncbi.nlm.nih.gov/pubmed/10918932. Accessed 6 June 2013. 10918932

[2] Wainberg MA, Friedland G (1998) Public health implications of antiretroviral therapy and HIV drug resistance. JAMA 279: 1977–1983. Available: http://www.ncbi.nlm.nih.gov/pubmed/9643862. Accessed 6 June 2013. 10.1001/jama.279.24.1977 9643862

[3] Altice FL, Friedland GH (1998) The era of adherence to HIV therapy. Ann Intern Med 129: 503–505. Available: http://www.ncbi.nlm.nih.gov/pubmed/9735090. Accessed 6 June 2013. 10.7326/0003-4819-129-6-199809150-00015 9735090

[4] Szakacs TA, Wilson D, Cameron DW, Clark M, Kocheleff P, et al. (2006) Adherence with isoniazid for prevention of tuberculosis among HIV-infected adults in South Africa. BMC Infect Dis 6: 97. Available: http://www.pubmedcentral.nih.gov/articlerender.fcgi?artid=1513236&tool=pmcentrez&rendertype=abstract. Accessed 6 June 2013. 10.1186/1471-2334-6-97 PMC1513236 16772037

[5] Balfour L, Kowal J, Silverman A, Tasca GA, Angel JB, et al. (2006) A randomized controlled psycho-education intervention trial: Improving psychological readiness for successful HIV medication adherence and reducing depression before initiating HAART. AIDS Care 18: 830–838. Available: http://www.ncbi.nlm.nih.gov/pubmed/16971295. Accessed 6 June 2013. 10.1080/09540120500466820 16971295

[6] Parienti J-J, Bangsberg DR, Verdon R, Gardner EM (2009) Better adherence with once-daily antiretroviral regimens: a meta-analysis. Clin Infect Dis 48: 484–488. Available: http://www.pubmedcentral.nih.gov/articlerender.fcgi?artid=2708315&tool=pmcentrez&rendertype=abstract. Accessed 21 May 2013. 10.1086/596482 PMC2708315 19140758

[7] Boyle BA, Jayaweera D, Witt MD, Grimm K, Maa J, et al. (2008) Randomization to once-daily stavudine extended release/lamivudine/efavirenz versus a more frequent regimen improves adherence while maintaining viral suppression. HIV Clin Trials 9: 164–176. Available: http://www.ncbi.nlm.nih.gov/pubmed/18547903. Accessed 6 June 2013. 10.1310/hct0903-164 18547903

[8] Friedman LM, Furberg CD, DeMets DL (2010) Fundamentals of Clinical Trials. 4th ed. New York: Springer.

[9] Baum M, Cassetti L, Bonvehi P, Shor-Posner G, Lu Y, et al. (1994) Inadequate dietary intake and altered nutrition status in early HIV-1 infection. Nutrition 10: 16–20. Available: http://www.ncbi.nlm.nih.gov/pubmed/8199417. Accessed 6 June 2013. 8199417

[10] Shevitz AH, Knox TA (2001) Nutrition in the era of highly active antiretroviral therapy. Clin Infect Dis 32: 1769–1775. Available: http://www.ncbi.nlm.nih.gov/pubmed/11360219. Accessed 6 June 2013. 10.1086/320761 11360219

[11] Marston B, De Cock KM (2004) Multivitamins, nutrition, and antiretroviral therapy for HIV disease in Africa. N Engl J Med 351: 78–80. Available: http://www.ncbi.nlm.nih.gov/pubmed/15229312. Accessed 6 June 2013. 10.1056/NEJMe048134 15229312

[12] Tang AM, Lanzillotti J, Hendricks K, Gerrior J, Ghosh M, et al. (2005) Micronutrients: current issues for HIV care providers. AIDS 19: 847–861. Available: http://www.ncbi.nlm.nih.gov/pubmed/15905665. Accessed 6 June 2013. 10.1097/01.aids.0000171398.77500.a9 15905665

[13] Irlam JH, Visser MM, Rollins NN, Siegfried N (2010) Micronutrient supplementation in children and adults with HIV infection. Cochrane Database Syst Rev: CD003650. Available: http://www.ncbi.nlm.nih.gov/pubmed/21154354. Accessed 22 May 2013. 10.1002/14651858.CD003650.pub2 16235333

[14] Baylin A, Villamor E, Rifai N, Msamanga G, Fawzi WW (2005) Effect of vitamin supplementation to HIV-infected pregnant women on the micronutrient status of their infants. Eur J Clin Nutr 59: 960–968. Available: http://www.ncbi.nlm.nih.gov/pubmed/15956998. Accessed 6 June 2013. 10.1038/sj.ejcn.1602201 15956998

[15] Jiamton S, Pepin J, Suttent R, Filteau S, Mahakkanukrauh B, et al. (2003) A randomized trial of the impact of multiple micronutrient supplementation on mortality among HIV-infected individuals living in Bangkok. AIDS 17: 2461–2469. Available: http://www.ncbi.nlm.nih.gov/pubmed/14600517. Accessed 6 June 2013. 10.1097/00002030-200311210-00008 14600517

[16] Austin J, Singhal N, Voigt R, Smaill F, Gill MJ, et al. (2006) A community randomized controlled clinical trial of mixed carotenoids and micronutrient supplementation of patients with acquired immunodeficiency syndrome. Eur J Clin Nutr 60: 1266–1276. Available: http://www.ncbi.nlm.nih.gov/pubmed/16721396. Accessed 6 June 2013. 10.1038/sj.ejcn.1602447 16721396

[17] Singhal N, Fergusson D, Huff H, Mills EJ, la Porte C, et al. (2010) Design and methods of the MAINTAIN study: a randomized controlled clinical trial of micronutrient and antioxidant supplementation in untreated HIV infection. Contemp Clin Trials 31: 604–611. Available: http://www.ncbi.nlm.nih.gov/pubmed/20708714. Accessed 6 June 2013. 10.1016/j.cct.2010.08.003 20708714

[18] Balfour L, Tasca G a, Kowal J, Corace K, Cooper CL, et al. (2007) Development and validation of the HIV Medication Readiness Scale. Assessment 14: 408–416. Available: http://www.ncbi.nlm.nih.gov/pubmed/17986658. Accessed 18 April 2013. 10.1177/1073191107304295 17986658

[19] DiMatteo MR, Hays RD, Sherbourne CD (1992) Adherence to cancer regimens: implications for treating the older patient. Oncology (Williston Park) 6: 50–57. Available: http://www.ncbi.nlm.nih.gov/pubmed/1532737. Accessed 6 June 2013. 1532737

[20] Bland JM, Altman DG (1997) Cronbach’s alpha. BMJ 314: 572. Available: http://www.pubmedcentral.nih.gov/articlerender.fcgi?artid=2126061&tool=pmcentrez&rendertype=abstract. Accessed 22 October 2013.

[21] Clark LA, Watson D (1995) Constructing validity: Basic issues in objective scale development. Psychol Assess 7: 309–319. Available: http://www.researchgate.net/publication/232454728_Constructing_validity_Basic_issues_in_objective_scale_development. Accessed 22 October 2013.

[22] Canadian Health Measures Survey: Cycle 2 Data Tables - 82-626-x2013001-eng.pdf (2013). Available: http://www.statcan.gc.ca/pub/82-626-x/82-626-x2013001-eng.pdf.

[23] Farley J, Hines S, Musk A, Ferrus S, Tepper V (2003) Assessment of adherence to antiviral therapy in HIV-infected children using the Medication Event Monitoring System, pharmacy refill, provider assessment, caregiver self-report, and appointment keeping. J Acquir Immune Defic Syndr 33: 211–218. Available: http://www.ncbi.nlm.nih.gov/pubmed/12794557. Accessed 23 October 2013. 10.1097/00126334-200306010-00016 12794557

[24] Van Servellen G, Chang B, Garcia L, Lombardi E (2002) Individual and system level factors associated with treatment nonadherence in human immunodeficiency virus-infected men and women. AIDS Patient Care STDS 16: 269–281. Available: http://www.ncbi.nlm.nih.gov/pubmed/12133262. Accessed 23 October 2013. 10.1089/10872910260066705 12133262

[25] Osterberg L, Blaschke T (2005) Adherence to medication. N Engl J Med 353: 487–497. Available: http://www.ncbi.nlm.nih.gov/pubmed/16079372. Accessed 23 October 2013. 10.1056/NEJMra050100 16079372

[26] Allard JP, Aghdassi E, Chau J, Tam C, Kovacs CM, et al. (1998) Effects of vitamin E and C supplementation on oxidative stress and viral load in HIV-infected subjects. AIDS 12: 1653–1659. Available: http://www.ncbi.nlm.nih.gov/pubmed/9764785. Accessed 6 June 2013. 10.1097/00002030-199813000-00013 9764785

[27] Semba RD, Ricketts EP, Mehta S, Netski D, Thomas D, et al. (2007) Effect of micronutrients and iron supplementation on hemoglobin, iron status, and plasma hepatitis C and HIV RNA levels in female injection drug users: a controlled clinical trial. J Acquir Immune Defic Syndr 45: 298–303. Available: http://www.ncbi.nlm.nih.gov/pubmed/17414930. Accessed 6 June 2013. 10.1097/QAI.0b013e318050d698 17414930

[28] Golin CE, Liu H, Hays RD, Miller LG, Beck CK, et al. (2002) A prospective study of predictors of adherence to combination antiretroviral medication. J Gen Intern Med 17: 756–765. Available: http://www.pubmedcentral.nih.gov/articlerender.fcgi?artid=1495120&tool=pmcentrez&rendertype=abstract. Accessed 6 June 2013. 10.1046/j.1525-1497.2002.11214.x PMC1495120 12390551

[29] Sheehan NL, van Heeswijk RPG, Foster BC, Akhtar H, Singhal N, et al. (2012) The effect of β-carotene supplementation on the pharmacokinetics of nelfinavir and its active metabolite M8 in HIV-1-infected patients. Molecules 17: 688–702. Available: http://www.ncbi.nlm.nih.gov/pubmed/22241465. Accessed 16 May 2013. 10.3390/molecules17010688 PMC6268962 22241465

