# Supplementary material for: Micronutrient Deficiency and Treatment Adherence in a Randomized Controlled Trial of Micronutrient Supplementation in ART-Naïve Persons with HIV
Source: PLoS One. 2014 Jan 21;9(1):e85607. doi: 10.1371/journal.pone.0085607 (PMC3897458; doi:10.1371/journal.pone.0085607)
Supplement: File S1 — The MAINTAIN Study Group. Full list of investigators, study coordinators, and data management personnel participating in the MAINTAIN study. (DOCX) [file pone.0085607.s002.docx]

**S1.The MAINTAIN Study Group.**

Louise Balfour^1,21^, Johanna N. Spaans^1,2,^, Dean Fergusson^2^, Harold Huff^4^, Edward J Mills^3^, Charles J la Porte^21^, Sharon Walmsley^1,8^, Neera Singhal^1,2^, Ron Rosenes^20^, Nancy Tremblay^1,21^, D. William Cameron^1,2,21^, M. John Gill^1,5^, Hugues Loemba^1,3^, Brian Conway^1,6^, Anita Rachlis^1,8^, Edward Ralph^1,7^, Mona Loutfy^1,8^, Richard Lalonde^1,12^, David Haase^1,9^, Roger Sandre^1,18^, Sylvie Trottier^1,11^, Wendy Wobeser^1,10^, Mark Hull^1,6^, Corinna Quan^1,17^, Danielle Rouleau^1,13^, Ken Kasper^1,15^, Emily Toma^1,14^, Fiona Smaill^1,16^, Bayan Missaghi^1,19^, Brenda Bechthold^1,5^, Vicky Lavazos^1,12^, Jennifer Lalonde^1,3^, Leala Wong^1,6^, Ania McNeil^1,8^, Michele Ellis^1,7^, Heather Haldane^1,9^, Judy Latendre-Paquette^1,18^, Rosie Clarke^1,8^, Isabelle Chabot^1,11^, Jenna Ekborn^1,10^, Marcela Gil^1,6^, Nancy McFarland^1,17^, Melissa Bonnetsmueller^1,21^, Chantal Morrisseau^1,13^, Heather Duckworth^1,15^, Lise Cyr^1,14^, Jennifer Hills^1,8^, Laura Puri^1,16^, Allyson Ion^1,16^, Kimberly Burt^1,19^, Jim Pankovich^1^ and David Cox^1^

^1^CIHR Canadian HIV Trials Network (CTN)

^2^The Ottawa Hospital Research Institute, Ottawa, Ontario, Canada

^3^The University of Ottawa, Ottawa, Ontario, Canada

^4^Canadian College of Naturopathic Medicine, Toronto, Ontario, Canada

^5^University of Calgary, Calgary, Alberta, Canada

^6^University of British Columbia, Vancouver, British Columbia, Canada

^7^University of Western Ontario, London, Ontario, Canada

^8^University of Toronto, Toronto, Ontario, Canada

^9^Dalhousie University, Halifax, Nova Scotia, Canada

^10^Queen’s University, Kingston, Ontario, Canada

^11^Centre Hospitalier Universitaire de Quebec (CHUQ), Quebec City, Quebec, Canada

^12^McGill University, Montreal, Quebec, Canada

^13^CHUM Notre Dame Hospital, Montreal, Quebec, Canada

^14^CHUM Hotel Dieu Hospital, Montreal, Quebec, Canada

^15^University of Manitoba, Winnipeg, Manitoba, Canada

^16^McMaster University, Hamilton, Ontario, Canada

^17^Windsor Regional Hospital, Windsor, Ontario, Canada

^18^Hôpital régional de Sudbury Regional Hospital, Sudbury, Ontario, Canada

^19^St. Clare’s Mercy Hospital, St. John’s, Newfoundland, Canada

^20^Canadian Treatment Action Council, Toronto, Ontario, Canada

^21^University of Ottawa at The Ottawa Hospital, Ottawa, Ontario, Canada
