# Supplementary material for: Micronutrient Deficiency and Treatment Adherence in a Randomized Controlled Trial of Micronutrient Supplementation in ART-Naïve Persons with HIV
Source: PLoS One. 2014 Jan 21;9(1):e85607. doi: 10.1371/journal.pone.0085607 (PMC3897458; doi:10.1371/journal.pone.0085607)
Supplement: Protocol S1 — (PDF) [file pone.0085607.s003.pdf]

Of the protocol at the STUDY PROTOCOL CTN 238

A RANDOMIZED CONTROL CLINICAL TRIAL OF MICRONUTRIENT & ANTIOXIDANT SUPPLEMENTATION IN PERSONS WITH UNTREATED HIV INFECTION

The MAINTAIN Study

Principal Investigator:
D. William CAMERON MD, FRCPC
Professor, Department of Medicine, Division of Infectious Diseases
University of Ottawa at The Ottawa Hospital, General Campus, Box 228
501 Smyth Road
Ottawa, Canada, K1H 8L6
Telephone: (613)-737-8923; Fax: (613)-737-8925; E-mail: bcameron@ohri.ca

Investigators:
Dean FERGUSSON PhD, MHA
Senior Scientist, Ottawa Hospital Research Institute
Assistant Professor, Faculty of Medicine, Epidemiology and Community Medicine
University of Ottawa at The Ottawa Hospital, General Campus
501 Smyth Road
Ottawa, Canada, K1H 8L6
Phone: 613-737-8899 x78480; Fax: 613-739-6266; E-mail: dafergusson@ohri.ca

Hal HUFF BA, ND
Associate Professor, Clinical Education, Canadian College of Naturopathic Medicine
Chief Clinical Supervisor, Sherbourne Health Centre People Living with HIV/AIDS
Canadian College of Naturopathic Medicine
1255 Sheppard Ave E.
Toronto, Ontario, Canada, M2K 1E2
Phone: 416-498-1255 x392; E-mail: hhuff@ccnm.edu

Charles la PORTE PhD, PharmD
Associate Scientist, Molecular Medicine, Ottawa Hospital Research Institute
Assistant Professor, Department of Medicine
University of Ottawa at The Ottawa Hospital, General Campus, Mailbox 223
501 Smyth Road
Ottawa ON, K1H 8L6
Phone: 613-737-8899 x71893; Fax: 613-737-8696; E-mail: claporte@ohri.ca

Ed MILLS PhD
Research Scientist, BC Centre for Excellence in HIV/AIDS
Simon Fraser University, Assistant Professor, Global Health
4208 West 10th Avenue, Vancouver, British Columbia, Canada
Phone : 778-317-8530; E-mail: emills@sfu.ca

Neera SINGHAL MBBS, MS, MHA
Senior Research Associate, Ottawa Hospital Research Institute
Assistant Professor, Department of Medicine, Division of Infectious Diseases
University of Ottawa at The Ottawa Hospital, General Campus, Box 228
501 Smyth Road
Ottawa, Canada, K1H 8L6
Telephone: 613-737-8899 x71892; Fax: 613-737-8280; E-mail: nsinghal@ohri.ca

Sharon WALMSLEY MD, FRCP
Professor, University of Toronto, Department of Medicine, Division of Infectious Disease
Toronto General Research Institute, Senior Scientist
Toronto General Hospital, Eaton Wing, 13th Floor, Room 218
200 Elizabeth Street, Toronto, Ontario, M5G 2C4
Telephone (416) 340-3871; Fax (416) 595-5826; E-mail: sharon.walmsley@uhn.on.ca

Collaborator:
Ron ROSENES, MA
Canadian Treatment Action Council
1491 Dupont Street
Toronto, Ontario
M6P 3S2
Phone: 416-588-5335; ron@rosenes.com

This protocol was developed with support from Board Directed Funds from the Ontario HIV Treatment Network (OHTN). The Ottawa Hospital Research Institute (OHRI) is the sponsoring institution. The study is supported by co-sponsor CIHR Canadian HIV Trials Network (CTN) and a Reseraarch Operating Grant from the OHTN.

TABLE OF CONTENTS

i. LAY SUMMARY ... 4
ii. EXECUTIVE SUMMARY ... 5
iii. STUDY FLOWCHART ... 6
1. BACKGROUND ... 7
2. HYPOTHESES ... 8
3. OBJECTIVES ... 9
3.1. Primary Objectives ... 9
3.2. Secondary Objectives ... 9
4. OVERVIEW OF STUDY DESIGN ... 9
5. PARTICIPANTS ... 9
5.1. Inclusion Criteria ... 9
5.2. Exclusion Criteria ... 10
6. STUDY DURATION ... 10
7. STUDY METHODS ... 10
7.1. Enrollment and Screening ... 10
7.2. Randomization ... 10
7.3. Safety Considerations ... 11
7.4. Outcome Measures ... 11
7.5. Disease Progression Requirements ... 12
7.6. Evaluating and Reporting Adverse Events ... 12
7.7. Withdrawal from the Study ... 13
7.8. Concomitant Medications ... 13
7.9. Study Visits ... 13
7.10. Sample size and Statistical Considerations ... 15
8. STUDY MEDICATION FORMULATION ... 16
8.1. Overview ... 16
8.2. Dosage and Regimen ... 17
9. ADMINISTRATIVE CONSIDERATIONS ... 17
9.1. Confidentiality ... 17
9.2. Ethics Review and Informed consent ... 17
10. QUALITY ASSURANCE ... 18
10.1 Responsibilities of the Institution ... 18
10.2. Responsibilities of the Investigator(s) ... 18
10.3. Responsibilities of the Manufacturer ... 18
11. STUDY SCHEDULE ... 19
REFERENCES ... 20
APPENDICES ... 24

i. LAY SUMMARY

Infection with human immunodeficiency virus (HIV) causes decline in immunity or the ability to fight infection and progresses to acquired immunodeficiency disease (AIDS). Anti-HIV drug treatment has improved the prognosis of persons with HIV infection, but is expensive and potentially toxic. Low micronutrient levels occur in the blood even in early stages of HIV infection and increase risk of a poorer prognosis, but the role of micronutrient and antioxidant supplements in medical management of HIV/AIDS is not well defined. The proposed clinical trial aims to assess if supplementation of untreated HIV-infected adults with a micronutrient and antioxidant preparation (109 study participants) can delay disease progression or start of anti-HIV drug treatment compared with supplementation with standard multivitamins (109 participants). Participants will be treated for two years and followed quarterly in clinic for two years, starting anti-HIV drug treatment as needed according to current treatment guidelines. We will study changes in number of immune cells in the blood, tolerance of study medication, and indicators of safety, well being and health. We expect supplementation with study medication to delay disease progression or start of anti-HIV medication. If the findings are positive, the study has implications for health and health care savings.

ii. EXECUTIVE SUMMARY

Background: Antiretroviral therapy (ART) has improved the prognosis of persons with human immunodeficiency virus (HIV) infection, but is expensive and potentially toxic. Micronutrient deficiencies occur even in early stages of HIV infection and increase risk of morbidity, disease progression to acquired immunodeficiency syndrome (AIDS) and mortality, but the role of micronutrient antioxidant supplements in medical management of HIV/AIDS is not clear.

Objective: To determine if supplementation of untreated asymptomatic HIV-infected persons with a broad-spectrum micronutrient and antioxidant preparation will reduce the rate of decline of CD4 T lymphocyte count, or delay emergence of documented CDC-defined AIDS-defining illness, or start of ART compared to 100% recommended daily allowance (RDA) multivitamins and minerals, and is safe.

Study design: A prospective, randomized, controlled, double blind clinical trial of supplementation of 218 untreated asymptomatic HIV-infected adults with a micronutrient and antioxidant preparation or identical appearing RDA multivitamins and minerals for two years, with quarterly follow up in clinic for assessment of time from baseline to CD4 count <350 mm^3, or emergence of documented CDC-defined AIDS-defining illness, or start of ART.

Participants and sample size: 218 participants from clinics in Ontario and other participating centres of the CIHR Canadian HIV Trials Network (CTN).

Study duration: Three years, allowing one year for participant accrual and two years follow-up.

Eligibility criteria: The main eligibility criteria are:

- Asymptomatic HIV-infected adults at least 18 years of age
- CD4+ cells ≥375 and ≤750 cells/mm^3
- No previous ART (excluding less than seven days and perinatal transmission prophylaxis)

Study intervention: Oral supplementation with a broad spectrum micronutrient and antioxidant preparation (n=109) or identical appearing RDA multivitamins and minerals (n=109).

Primary outcome: Time from baseline to CD4+ cell count <350 cells/mm^3 (confirmed by two measures at least one week apart), or emergence of documented CDC-defined AIDS-defining illness, or start of ART

Secondary outcomes:

- Non-AIDS related adverse events
- Tolerance of and adherence to study medication
- Time from baseline to CD4+ cell count <350 cells/mm^3 (confirmed by two measures at least one week apart)
- Time from baseline to emergence of documented CDC-defined AIDS-defining illness
- Time from baseline to start of ART
- Serial quarterly lymphocyte measures: absolute lymphocyte count (ALC), CD4+, CD8+, and CD3+ cell counts, CD4%, CD8%, CD4:CD8
- Serial quarterly HIV RNA plasma viral load
- Serum chemistries: Glucose, BUN, creatinine, total protein, C-reactive protein, albumin, alkaline phosphatase, ALT, AST, total bilirubin
- Serum micronutrient levels: Carotene (quarterly) and vitamin B_{12} (quarterly), folate (six monthly) and vitamin D (25-OHD, six monthly)
- Quality of Life measures: MOS HIV, EuroQol, and Health Utilities Index (HUI)

Statistical analysis: Analysis of the primary outcome by intention-to-treat will compare time from baseline to primary outcome. Interim analyses are planned once 100 participants are followed for one year.

iii. STUDY FLOWCHART

218 consenting eligible participants will be enrolled during routine clinic visits and randomized to two groups
Neither research staff nor participants will know assigned treatment

↓ ↓

Treatment group	Control group
n=109	n=109
Will receive daily a micronutrient and antioxidant preparation for two years	Will receive identical appearing standard multivitamins

↓ ↓

Participants will be followed in quarterly clinic visits for two years
They will be offered opportunistic infection prophylaxis and begin ART as indicated according to current treatment guidelines

↓ ↓

On reaching primary outcome (CD4+ cells $<350/mm^3$, or CDC-defined AIDS-defining illness, or start of ART), participants in both groups will be offered open label study medication with continued follow up until two years total, but censored from analysis

↓ ↓

Analyses of primary outcome by intention-to-treat of all participants randomized will compare time from baseline to primary outcome between groups
Secondary outcomes relate to indicators of safety, well being and health

1. BACKGROUND

Low serum levels of micronutrients are common in persons with human immunodeficiency virus (HIV) infection and acquired immunodeficiency syndrome (AIDS), even in early stages of infection[1-6], and are associated with morbidity and mortality, disease progression, and mother-to-child viral transmission[7-12]. Besides low dietary intake, low micronutrient concentration in HIV-infected persons may be due to malabsorption, diarrhea, altered gut barrier function, and altered metabolism[13], in addition to the depletion produced by increased levels of oxidative stress[14-17]. While micronutrient deficiencies are to be expected in poor countries, they also occur in developed countries[5], where dietary intake by HIV-infected individuals is adequate and un-prescribed micronutrient use is common[4]. Despite the association between low micronutrient levels and poor clinical outcomes in HIV-infected adults, clinical trials of micronutrient supplementation show no conclusive evidence of benefit of micronutrients on morbidity and mortality and on mother-to-child viral transmission, although some but not all studies show some clinical and immunological benefit[18-20]. Indefinite antiretroviral therapy (ART) prolongs life, but is expensive and toxic.

In a 12-week placebo controlled trial, micronutrient and antioxidant supplementation with the proposed preparation showed a significant 24% increase in CD4 T lymphocytes in patients on a stable highly active antiretroviral therapy (HAART) regimen[21], while without antioxidant supplementation immunologic benefit is modest and slower[22,23]. In laboratory experiments and in clinical studies, the antioxidants acetyl L-carnitine (LAC), N-acetyl-cysteine (NAC) and alpha-lipoic acid have each shown the potential of beneficial effects in HIV infection[24-32]. In a randomized placebo-controlled clinical trial in 481 ART-untreated adults with HIV infection, supplementation with micronutrients showed a salutary effect on survival, with no accompanying changes in T lymphocyte counts[33]. In a large randomized placebo-controlled factorial clinical trial in ART-untreated pregnant women with HIV infection, supplementation with vitamins B, C, and E but not vitamin A showed an increase in CD4 and CD8 T lymphocyte counts and delay in the progression of HIV disease when compared to placebo[34,35].

β-carotene supplementation enhances cellular immunity[36]. In a cross-sectional study in HIV-1 seropositive adults, low serum concentrations of beta-carotene correlated with markers of HIV infection[37]. In a controlled clinical trial in 331 AIDS patients, low serum carotene was associated with mortality and supplementation with natural mixed carotenoids and multivitamins and minerals was associated with improved survival compared with multivitamins and minerals alone[38]. Supplementation with vitamins B, C, and E but not vitamin A may be associated with reduction in viral load[35]. Deficiency of vitamin$_6$ is prevalent in HIV-1 infected persons despite adequate dietary vitamin B_6 intake[39] and has been associated with immunological changes[40]. Vitamin B_{12} deficiency is prevalent in HIV infection[41] and associated with HIV disease progression[42].

Supplementation with the antioxidant LAC was associated with a decrease in CD4 T lymphocyte apoptosis in both symptomatic[24] and asymptomatic[25] zidovudine and didanosine treated HIV-infected patients. In a study of oral supplementation of HIV-infected neuropathic patients with LAC for 33 months, distal symmetrical polyneuropathy symptoms improved, accompanied by significant nerve fiber regeneration[31].

Laboratory studies show that NAC is associated with rescuing of peripheral blood lymphocytes from apoptosis [29], by protection from cyclohexamide and necrosis factor-alpha (TNF)[30]. Sulfur is lost in HIV-infected patients as a consequence of peripheral tissue cysteine catabolism and this loss is not ameliorated by HAART[43]. Supplementation of NAC in HIV-infected adults in placebo-controlled randomized clinical trials was associated with a significant increase in

immunological function[26,27] and in exposed HIV-negative individuals to increased release of chemokines that may decrease susceptibility to HIV infection[28].

Alpha lipoic acid has a dose dependent inhibition of HIV-1 replication in cytopathic effect formation, reverse transcriptase activity and plaque formation on CD4-transformed HeLa-cells and no toxic effect on blood mono-nuclear cells and T-cell lines[32]. Micronutrient interactions may also be associated with reduced morbidity of infections[44] and improved response to drugs[45].

We propose a clinical trial to assess if micronutrient and antioxidant supplementation in untreated asymptomatic HIV-infected persons will reduce the rate of decline in CD4 T lymphocyte count, or emergence of CDC-defined[46] AIDS-defining illness, or start of ART versus recommended daily allowance (RDA) multivitamins and minerals. Since multivitamins are commonly used by HIV-infected persons[4] and it is not our intent to evaluate study medication in the absence of multivitamins, participants in the control arm will receive RDA multivitamins and minerals. In our previous clinical trial of mixed carotenoids supplementation also, both the treatment and control arms received RDA multivitamins and minerals[38].

Safety of the proposed medication

The proposed medication has been used safely for three months in HIV-infected individuals[21] and is sold over the counter in the United States. Other longer studies of micronutrient and antioxidant supplementation in comparable dosage in HIV-infected and -uninfected adults show no harm[38,47]. A meta-analysis of clinical trials shows no harm of antioxidant supplementation, except possible increased mortality with some antioxidants in a secondary analysis[48]. A possible adverse effect of β-carotene in smokers and those exposed to asbestos[49], and with high intake in HIV-infected adults in an observational study[50] have not been confirmed in large randomized studies. Carotenemia and skin discoloration, both reversible conditions, have not been reported in HIV infection even with beta-carotene doses higher than proposed here. In non-HIV individuals, flushing of the skin that is generally mild and transient occasionally occurs with nicotinic acid (vitamin B_3) at a daily dose greater than 10 mg. Supplementation of nucleoside reverse transcriptase inhibitor-treated HIV-infected adults with vitamins C and E and NAC was associated with increased fasting glucose levels at 24 weeks, but the study was small and uncontrolled and all the participants either had lipoatrophy or sustained hyperlactatemia[51]. These and other micronutrients and antioxidants were well tolerated in other studies[21,38]. There is no evidence of harm of iron supplementation in HCV co-infected HIV-infected adults[52]. Micronutrient and iron supplementation of coinfected female injection drug users improves anemia without increasing plasma HCV or HIV RNA levels or altering liver enzymes[53], and no harm has ever been shown at the proposed iron dosage of 18 mg. Hepatitis coinfection patients are not excluded from the study as they are more likely to be nutrient depleted and oxidative stressed and could be helped by the intervention.

2. HYPOTHESES

Primary: Supplementation of untreated asymptomatic HIV-infected persons with broad spectrum micronutrients and antioxidants will delay decline from baseline in CD4 T lymphocyte count to <350 cells/mm^3, or emergence of CDC-defined AIDS-defining illness[46], or start of ART compared with 100% RDA multivitamins and minerals.

Secondary: Supplementation of untreated asymptomatic HIV-infected persons with broad spectrum micronutrients and antioxidants will be well tolerated and safe.

3. OBJECTIVES

3.1 Primary Objective

- To evaluate if supplementation of untreated asymptomatic HIV-infected adults with micronutrients and antioxidants will reduce the rate of decline of CD4 T lymphocyte count, or delay emergence of CDC-defined AIDS-defining illness[46], or delay start of ART versus RDA multivitamins and minerals.

3.2 Secondary Objectives

- To evaluate safety and adverse events
- To evaluate if study medication is well tolerated
- To evaluate effect of study medication on T lymphocyte immunophenotype subsets
- To evaluate effect of study medication on emergence of CDC-defined AIDS defining illness
- To evaluate if study medication can delay initiation of ART
- To evaluate effect of study medication on plasma HIV RNA viral load
- To evaluate effect of study medication on health related quality of life

4. OVERVIEW OF STUDY DESIGN

This will be a prospective randomized, controlled, double blind, multi-centre clinical trial of supplementation of 218 untreated asymptomatic HIV-infected consenting, eligible participants with a micronutrient and antioxidant preparation or 100% RDA multivitamins and minerals to assess time from baseline to CD4 T lymphocyte count <350 cells/mm^3, or emergence of CDC-defined AIDS-defining illness[46], or start of ART. Patients will be approached during routine clinic visits, and consenting participants screened. Eligible participants will be randomized to one of two equal study groups to receive the study or control medications for two years and be followed during quarterly clinic visits for two years. Participants will be offered opportunistic infection prophylaxis. They will begin ART as and when indicated according to current treatment guidelines (2008)[54], with indicated changes to the ART regimen permitted. The primary outcome measure is time from baseline to CD4 T lymphocyte count <350 cells/mm^3, or emergence of documented CDC-defined AIDS-defining illness[46], or start of ART. Secondary outcomes include tolerance of study medication and indicators of safety, well being, and health. Analyses of the primary outcome will be by intention-to-treat of all participants randomized to the intervention, comparing outcome measures between study groups. Once participants in either group reach outcome (CD4 T lymphocyte count <350 cells/mm^3, or emergence of CDC-defined AIDS-defining illness[46], or start of ART), they will be offered open label study medication with continued quarterly follow up, but censored from analysis.

5. PARTICIPANTS

Study participants will be from CIHR Canadian HIV Trials Network (CTN) centres in Ontario and across Canada.

5.1 Inclusion Criteria

The participant must:

- be an asymptomatic HIV infected adult
- at least 18 years of age

- have CD4+ cells ≥375 and ≤750 cells/mm^3
- have received no ART (excluding less than seven days and perinatal transmission prophylaxis)
- if a woman of child bearing potential, have a negative pregnancy test within two weeks prior to randomization and agree to practice barrier method of birth control during the study
- be willing and able to sign informed consent and to comply with the study protocol

5.2 Exclusion Criteria

The participant must not:

- have HIV-2 infection alone
- have known allergy or intolerance to any study medication ingredient
- be pregnant
- have active treatment for an acute opportunistic infection or malignancy
- have ALT greater than 3 x normal range
- have known cirrhosis of the liver
- have serum creatinine >133 µmol/L
- abuse alcohol and recreational drugs
- be taking micronutrient (except vitamin D*) or natural health product supplements within 30 days of randomization

*Maximum 2000 IU daily (Health Canada Guidelines).

6. STUDY DURATION

We expect the study to last three years, with accrual of participants requiring approximately 12 months and two years follow up of the last enrolled participant.

7. STUDY METHODS

7.1 Enrollment and Screening

Participants will be enrolled in the study during routine clinic visits. Research staff will do a preliminary screening for eligibility, explain the clinical trial to the potential participant, ask them to read and sign an information sheet and consent form, and screen for eligibility to participate in the study.

7.2 Randomization

Each participant will receive a unique Participant Identification (ID) number. A permuted blocked randomization method, stratified by centre and CD4+ cell count balanced above and below 500 cells/mm^3, will be used to allocate participants to study group. An independent biostatistician from the Ottawa Methods Centre will generate the randomization scheme. The randomization process will consist of a computer-generated random listing of the treatment allocations stratified by centre and with CD4+ cell count balanced equally above and below 500 cells/mm^3 in variable permuted blocks of 2 and 4. The randomization scheme output will be given to The Ottawa Hospital (TOH) Pharmacy in an unblinded fashion. Personnel at TOH Pharmacy will prepare the medication kits and assign a participant ID number to each kit, and will send labeled kits to sites. The system will have backup in the form of a statistician and designate at the coordinating centre. Only the study statistician and designate and designated pharmacist(s) at the coordinating centre will have knowledge of treatment allocation. Sites will access the password protected MAINTAIN Study Web Randomization site to obtain the Participant ID number after screening.

Procedure for users (sites) to obtain Participant ID number from MAINTAIN Randomization Web site
After screening the participant for eligibility and obtaining informed consent, the site research nurse (user) will assign the participant a screening number. The user will login to the password protected Web system, and the system will ask a series of questions. The system will take the user to their specific site, and will ask for the participant screening number. The system will check to ensure the screening number is not used already. Users will then be asked if the participant's CD4+ cell count is below 500 cells/mm^3 or if it is 500 or greater. The system will then assign blinded randomization number (Participant ID number) for that participant, and the user will print results and log off the Web system. The participant will receive the pharmacy kit matching their participant ID number.

Methods for protecting against other sources of bias
In order to minimize selection and ascertainment biases, physicians, nurses, investigators, and research staff will be blinded to the randomization schemes and treatments administered. The trial statistician will designate another statistician to prepare all randomization schemes and interim analyses. Only a designated research pharmacist(s) at the coordinating centre will be aware of the treatment allocation for individual participants. The research pharmacist(s) will not have contact with the study teams or participants and will be expressly forbidden to discuss individual treatment allocation with the clinical care teams. To help ensure an effective masking of treatments, we will test whether physicians, research nurses, and participants can physically distinguish the treatment medication from control RDA multivitamins and minerals. This will be conducted before the start of the trial. At the end of the follow-up period, we will ask investigators, outcome assessors, and participants their hunches as to what treatment they received.

7.3 Safety Considerations

Adverse events attributable to the intervention and laboratory parameters of safety will be assessed and monitored by the Data Safety and Monitoring Board (DSMB). If there is concern for safety as a result of the study, or due to new information regarding the study formulation or its components, the Study Steering Committee will make a decision regarding dissemination of the information to the participants. If new information regarding the study or the study medication or its components raises concerns for the safety of the participants, the Study Steering Committee will defer the decision to CTN's Safety and Efficacy Review Committee (SERC), an autonomous body with authority to discontinue the study. The DSMB and all study committees will include a community representative.

7.4 Outcome Measures

Primary

- Time from baseline to CD4+ cell count <350 cells/mm^3 (confirmed by two measures at least one week apart), or emergence of documented CDC-defined AIDS-defining illness[46]*, or start of ART

  To accommodate biological and systematic variation and confirm the CD4+ cell count measure, each participant will have two measures of CD4+ cell count at least one week apart at baseline (and up to six weeks apart), at outcome (CD4+ cell count <350 cells/mm^3), and at end of study (Week 96 or premature withdrawal). CD4+ cell counts will be done in clinical laboratories

participating in Canadian CD4+ standardization program with the National HIV Laboratory (Ottawa).

Secondary

- Non-AIDS related adverse events
- Tolerance of and adherence to study medication
- Time from baseline to CD4+ cell count <350 cells/mm^3 (confirmed by two measures at least one week apart)
- Time from baseline to emergence of documented CDC-defined AIDS-defining illness[46]*
- Time from baseline to start of ART
- Serial quarterly lymphocyte measures: absolute lymphocyte count (ALC), CD4+, CD8+, and CD3+ cell counts, CD4%, CD8%, CD4:CD8
- Serial quarterly HIV RNA plasma viral load
- Serum chemistries: Glucose, BUN, creatinine, total protein, C-reactive protein, albumin, alkaline phosphatase, ALT, AST, total bilirubin
- Serum micronutrient levels: Carotene (quarterly), vitamin B_{12} (quarterly), folate (six monthly) and vitamin D (25-OHD, six monthly)
- Quality of Life measures: MOS HIV, EuroQol, and Health Utilities Index (HUI)
  *See Appendix A (oral thrush is not considered an AIDS-defining illness)

Study outcomes will be measured by procedures at study visits, described in section 7.9 and Table 1 (Section 11).

Efficacy will be measured by assessment of the primary outcome of time from baseline to reduction of CD4+ cell count below 350 cells/mm^3, or emergence of AIDS-defining illness, or start of ART. Secondarily, efficacy will be measured by assessing each component of the primary outcome, T lymphocyte immunophenotype subsets, plasma viral load, micronutrient levels, and validated quality of life questionnaires. Micronutrient levels measurements will assess baseline deficiency and its correction with treatment. *Adherence* will be measured by pill count, the validated HIV Adherence Treatment Scale (HATS) questionnaire[55], and serial measurement of serum carotene, which is a measure of intestinal absorption. *Safety* will be measured by adverse events, urinalysis, hematology and serum chemistries.

7.5 Disease Progression Requirements

If the participant has experienced any new or recurrent documented HIV-related or CDC-defined AIDS-defining illness[46] (see Appendix A) since the last visit, the Investigator/study coordinator is required to complete the HIV Disease Progression section of the case report forms (CRF). The HIV-related and AIDS-defining illnesses need not be reported in the Adverse Event section.

7.6 Evaluating and Reporting Adverse Events

7.6.1. Adverse Events

The Investigator or designate must complete the Adverse Event Record Form if the participant has experienced a new event or change in existing medical condition or symptom that is non-HIV related, and is either observed or volunteered, serious or non-serious. The reporting of adverse events will be elicited by non-leading questions. The Investigator will designate and document each adverse event as being mild, moderate or severe in nature, and estimate the possible relationship of the event to study medication. The Investigator must record a detailed description of the event with

onset and resolution dates and times, an indication of its severity, and any action taken as a consequence in the CRF. Appropriate procedures to investigate any adverse event may be initiated at the discretion of the Investigator. All adverse events must be followed until they are fully explained or until they have resolved or stabilized.

7.6.2. Serious or Unexpected Adverse Event Reporting

The Investigator shall make an accurate and adequate special report to the coordinating centre (OHRI),and to any Institutional Review Board/Research Ethics Board (IRB/REB) that has reviewed and is continuing to review the investigation, on any serious adverse events. All serious and/or significant unexpected adverse events that occur during the study or post-therapy period, regardless of treatment group or relationship to medication, MUST BE REPORTED WITHIN 24 HOURS BY TELEPHONE TO THE COORDINATING CENTRE. The coordinating centre must receive an initial report of all serious adverse events as soon as practically possible and a written report within 72 hours. All reports of death or life-threatening adverse events must be reported as soon as possible and no later than seven working days to the aforementioned parties.

7.7 Withdrawal from the Study

Participants may withdraw from the study at any time and for any reason, however, they will be urged to stay on their assigned medications in the absence of illness. The coordinating centre (OHRI) or the Investigator may also withdraw the participant at their discretion, or in the event of intercurrent illness, intolerance to study formulation, adverse events, pregnancy, protocol violation, or administrative reasons. All participants discontinued due to an adverse event must be followed until the event resolves, or becomes chronic or stable. Appropriate medical care must be provided until signs and symptoms have remitted, stabilized, or until abnormal laboratory findings have returned to acceptable or pre-study limits. Unnecessary withdrawal of participants should be avoided. If discontinued due to pregnancy, fetal outcome must be monitored, because of unknown potential risk to the fetus.

7.8 Concomitant Medications

While participating in the study and within 30 days of start of the study, the participant may not take another micronutrient (except vitamin D*) or natural health product, and alcohol intake will be limited to three drinks per day or about 50 gm. Illicit and recreational drugs must not be abused. The participant will be offered opportunistic illness prophylaxis. Antiretroviral therapy will be started and changes made as and when indicated according to current treatment guidelines (2008)[54].
*Daily vitamin D intake must not exceed 2000 IU (Health Canada guidelines).

7.9 Study Visits

Participants will come for study visits as described below. See Section 11 for Study Schedule. Laboratory tests will be conducted at or through the laboratory of each centre. Results of blood tests are acceptable within 30 days of the visit. Each participant will have two measures of CD4+ cell count at least one week apart at baseline (and up to six weeks apart), at outcome (CD4+ cell count <350 cells/mm^3) and at end of study (Week 96 or premature withdrawal). For CD4+ cell counts, the clinical laboratories must be participating in Canadian CD4+ cell standardization program with the National HIV Laboratory (Ottawa).

Pre-treatment evaluation (Screening Visit, Week -4 to -2)

The participant will be assessed for study eligibility by matching the results of the following procedures to eligibility criteria:

- Informed consent and demographics

- Medical history including previously diagnosed medical conditions, CDC-defined HIV-related or AIDS-defining illness, opportunistic infections, and previous and current medications (including any prior ART and their start and stop dates) and alcohol, illicit and recreational drug, and natural health product use
- Complete physical examination, including vital signs and signs and symptoms including of HIV disease
- Height and weight, for calculation of BMI
- HIV serology, if result not documented, send for testing
- HIV RNA (PCR)
- Lymphocyte measures: absolute lymphocyte count (ALC), CD4+, CD3+, and CD8+ cell counts, CD4%, CD8%, CD4:CD8
- Hematology: white blood cell (WBC) count with differential, red blood cell (RBC) count, hemoglobin, hematocrit, MCV, ESR, platelet count
- Coagulation test (INR)
- Serum chemistry: Glucose, blood urea nitrogen (BUN), creatinine, total protein, C-reactive protein. albumin, alkaline phosphatase, alanine transaminase (ALT), aspartate transaminase (AST), total bilirubin
- Pregnancy test: A urine pregnancy test will be performed at screening for all females able to bear a child. A β-HCG blood test will be performed for re-test of positive or equivocal urine result Urinalysis; microscopic examination will be performed only if indicated

Baseline Visit (Week 0)

The following procedures will be done at this visit:

- Height and weight, for calculation of BMI
- Update medical history (see screening visit) and confirm eligibility
- Subjective complaints or symptoms
- Medications, including over-the-counter and ART, and alcohol, illicit and recreational drug and natural health product use
- Targeted physical examination, including for objective findings, e.g., Weight change, fever, diarrhea, oral hairy leukoplakia, seborrheic dermatitis, oral candidiasis, lymphadenopathy, hepatosplenomegaly, and other documented HIV-related or CDC-defined AIDS-defining illness (see Appendix A)
- Adverse events (not related to study medication)
- Questionnaires (MOS-HIV, EuroQol, and Health Utilities Index (HUI))
- Hepatitis serology (HBV DNA or HBsAg) and (HCV RNA or HCV Ab); if results not documented, send for testing
- Haematology, consisting of WBC count with differential, RBC count, hemoglobin, hematocrit, MCV, ESR, platelet count
- Serum chemistry: Glucose, BUN, creatinine, total protein, C-reactive protein, albumin, alkaline phosphatase, ALT, AST, total bilirubin
- Serum micronutrient levels: carotene, vitamin B_{12}, vitamin D (25-OHD) and folate
- Coagulation test (INR)
- Lymphocyte measures: ALC, CD4+, CD3+, and CD8+ cell counts, CD4%, CD8%, CD4:CD8. CD4+ cell count confirmed by two measures at least one week and up to six weeks apart
- Plasma HIV RNA (PCR)
- Urinalysis; microscopic examination will be performed only if indicated

- Pregnancy test: A urine pregnancy test will be performed at baseline for all females able to bear a child. A β-HCG blood test will be performed for re-test of positive or equivocal urine result. Subsequently, a pregnancy test will be done only as needed (delayed menses)
- Dispense study medication

Follow up Quarterly Visits (Weeks 12, 24, 36, 48, 60, 72, 84, and 96)

Follow up visits may be scheduled within six days of the actual visit date

- Subjective complaints or symptoms
- Targeted physical examination including for objective findings, e.g., Weight change, fever, diarrhea, oral hairy leukoplakia, seborrheic dermatitis, oral candidiasis, lymphadenopathy, hepatosplenomegaly, and other documented HIV-related or CDC-defined AIDS-defining illness (see Appendix A). A complete physical examination must be done at end of study (Week 96 or premature withdrawal)
- Adverse events and their considered relationship to the study medication (if applicable)
- All adverse events and HIV/AIDS events to be followed until resolution or become stable
- Questionnaires (MOS-HIV, EuroQol, and Health Utilities Index (HUI))
- Weight, for calculation of BMI
- Residual pill count and HATS adherence questionnaire to assess adherence to study medication
- Medications, including over-the-counter and ART, and alcohol, illicit and recreational drug, and natural health product use
- Haematology, consisting of WBC count with differential, RBC count, hemoglobin, hematocrit, MCV, ESR, platelet count
- Serum chemistry: Glucose, BUN, creatinine, total protein, C-reactive protein, albumin, alkaline phosphatase, ALT, AST, total bilirubin
- Serum micronutrient levels: carotene (quarterly), vitamin B_{12} (quarterly), vitamin D (25-OHD, six monthly) and folate (six monthly)
- Coagulation test (INR)
- Lymphocyte measures: ALC, CD4+, CD3+, and CD8+ cell counts, CD4%, CD8%, CD4:CD8. CD4+ cell count confirmed by two measures at least one week apart at outcome (CD4+ cell count <350 cells/mm^3) and end of study (Week 96 or premature withdrawal)
- Plasma HIV RNA (PCR)
- Urinalysis; microscopic examination will be performed only if indicated
- Dispense study medication up to Week 84 visit

Study termination visit (two to six weeks after 96-week visit or premature withdrawal from the study)
All the procedures of a quarterly visit will be done.

7.10 Sample Size and Statistical Considerations

7.10.1 Sample Size Summary

Estimated time to CD4 count <350 cells/mm^3 or start of ART was based on previous research[56] and expert opinion. In the UK study, 45% of participants had either a CD4+ count below 350/mm^3 or started ART at 2 years and 60% met the outcome at 3 years[56]. In addition to the CD4+ count <350/mm^3 and start of ART estimates based on more than 25,000 patients, we will measure all observed CDC-defined AIDS-defining illness. As such, we have estimated that the control median time from baseline to CD4+ count <350 cells/mm^3, or emergence of documented CDC-defined

AIDS-defining illness, or start of ART to be 50% at 2 years. Based on our previous trial, and expert opinion and consensus, we hypothesized that treatment with micronutrients and antioxidants would decrease the median to 32% (18% absolute change in median at 2 years). A two-sided log rank test with an overall sample size of 218 participants (of which 109 are in the treatment group and 109 in the control group) achieves 80% power at a 0.05 significance level to detect a difference of 18% between the proportions surviving in the control and treatment groups after 2 years. Participants will be enrolled during an accrual period of one year and 50% of the enrollment will be complete when 50% of the accrual time has passed. We have also accounted for a modest non-compliance rate of 15% over the two years for those participants allocated to active treatment medication and 20% for those allocated to control. These estimates are based on consensus of opinion among investigators and the observations seen in our previous trials[38,57]. See Appendix B for detailed calculation.

7.10.2 Statistical Analysis

Principal analysis of primary outcome measure: All statistical analyses will be based on an intention-to-treat approach. All participants enrolled will be analyzed according to the intervention to which they were allocated, whether they received it or not. The primary outcome (CD4 count <350 cells/mm^3, or emergence of CDC-defined AIDS-defining illness, or start of ART) will be analyzed using a two-sided log-rank test. We will also conduct Cox-proportional hazards models to adjust for important prognostic risk factors and/or clinically important baseline imbalances. Participants who reach the primary outcome of confirmed CD4 T lymphocyte count <350 cells/ mm^3, or emergence of CDC-defined AIDS-defining illness, or start of ART will be censored.

Analysis of secondary outcome measure: Continuous outcomes including T cell measures (ALC, CD4+, CD4%, CD3+, CD8+ count, CD8%, CD4:CD8 ratio, HIV RNA plasma viral load), serum chemistry measures (such as ALT, creatinine, C-reactive protein, glucose, carotene, folate and vitamins B_{12} and D) will be analyzed using either parametric (independent t-test) or non-parametric procedures (Wilcoxon Rank Sum) followed by generalized linear regression models that adjust for important risk factors. Mean differences in Quality of Life measures between groups will be compared with a two-sample t-test if values are normally distributed. Alternatively, we will use a Mann Whitney test. Adverse events (not related to AIDS) and tolerance of medication will be compared using unadjusted chi-square tests.

Co-interventions, adherence and losses to follow-up: A number of secondary analyses will be performed to better understand the influence of co-interventions, adherence and losses to follow-up on the robustness of the intention-to-treat analysis. The general approaches will include: (a) an analysis of primary and secondary outcomes including only participants who completed the study as per protocol; (b) inclusion of co-interventions in all multivariate analyses; and (c) a best-case, worse-case scenario for losses to follow-up if they exceed 5% in either group.

Interim analyses are planned once 100 participants have been followed for one year.

8. STUDY MEDICATION FORMULATION

8.1 Overview

The micronutrient antioxidant preparation (K-PAX Ultra®) and identical appearing control medication will be supplied by K-PAX Inc. (USA). See Appendix C for composition.

Compassionate provision of study medication
If study results are positive, for one year after the study ends the manufacturer will make available study medication free to control arm participants and at discounted prices to all participants. Provision of the medication and discount will depend on obtaining regulatory approval from Health Canada, as K-PAX is not sold in Canada.

8.2 Dosage and Regimen

8.2.1 Treatment allocation
Treatment group (Group A): Will receive micronutrients and antioxidants.
Control group (Group B): Will receive identical appearing RDA multivitamins and minerals.

8.2.2 Dosage: The treatment and control medications will be taken as one packet (8 capsules) twice a day with meals. Because of the presence of calcium, iron and zinc in the study medication, any other medication must be taken at least two hours before or after taking it. Participants may initiate the intervention at half dose (one packet of 8 capsules once a day) and increase to full dose after one week.

8.2.3 Duration of the intervention
The intervention will last for two years. On reaching primary outcome (CD4+ cell count <350 cells/mm^3, or emergence of documented CDC-defined AIDS-defining illness, or start of ART), the participant may be switched to labeled (unblinded) study medication, irrespective of the assigned Group. The participant will continue to be followed for two years total, but will be censored from analysis.

9. ADMINISTRATIVE CONSIDERATIONS

9.1 Confidentiality

All information pertaining to the study, including pre-clinical data, product monograph, protocols, CRFs, and verbal and written information, will be kept strictly confidential and confined to the clinical personnel involved in conducting the study. This information may be relayed in confidence to the REB/IRB or appropriate regulatory authorities, except if required by law. CRFs and all other documents containing confidential participant information leaving the Investigator’s site will not identify the participant by name, but only by a code.

9.2 Ethics Review and Informed Consent

The study protocol and informed consent form were developed with the help of a Community Advisory Committee. Each participating site will obtain approval to conduct this study from their REB/IRB. Each site may make minor modifications to the sample Participant Information Sheet and Informed Consent Form according to the recommendations of their Ethical Review Committee. A copy of the protocol, including protocol amendments, all versions of informed consent forms, information to be completed by participants such as survey instruments or questionnaires must be reviewed and approved by the REB/IRB of each participating centre before implementation. The investigator will notify the REB/IRB of violations from the protocol and serious adverse events. The Investigator will provide a copy of the signed informed consent to the participant, and an original shall be maintained in the participant's medical record. This study will be conducted in accordance with the Therapeutic Products Directorate’s Good Clinical Practice (GCP) guidelines

(www.hc-sc.gc.ca/dhp-mps/prodpharma/applic-demande/guide-ld/ich/efficac/e6_e.html) and Health Canada Food & Drug Regulations, Part C Division 5: http://laws.justice.gc.ca/en/F-27/C.R.C.-c.870/index.html and in accordance with the current Declaration of Helsinki (http://www.wma.net/e/policy/b3.htm) and Code of Ethical Conduct for Research Involving Humans (http://www.ethics.ubc.ca/code/july97/). Appropriate reports on the progress of this study will be made by the Principal Investigator to the REB/IRB and the sponsor in accordance with applicable government regulations. Upon completion of the study and finalization of the study report, the Investigator must provide a summary to the REB/IRB.

10. QUALITY ASSURANCE

10.1 Responsibilities of the Institution

The monitoring of the study at each participating site will be in accordance with ICH Harmonized Tripartite Guidelines for Good Clinical Practice and Conduct of Clinical Investigations (Drugs Directorate, Health Canada) (www.hc-sc.gc.ca/dhp-mps/prodpharma/applic-demande/guide-ld/ich/efficac/e6_e.html). Quality control procedures will be implemented beginning with the data entry system and data quality control checks that will be run on the database generated. Any missing data or data anomalies will be communicated to the site(s) for clarification/resolution.

10.2 Responsibilities of the Investigator(s)

The Investigator undertakes to perform the study in accordance with GCP.

10.3 Responsibilities of the Manufacturer

There will be quality assurance of the study medication with frequent and uninterrupted re-supply. Contents of study medication have been shown to be stable (they were at or above label claim) at one year post-production and will be assayed every six months by the manufacturer.

11. STUDY SCHEDULE

Table 1: Study Schedule

PROCEDURES	STUDY VISITS		
	**Screen Weeks –4 to -2**	**Baseline Week 0**	**Weeks 12, 24, 36, 48, 60, 72, 84, and 96 Weeks and Study Termination Visit***
Informed consent and demographics	x		
Medical history**	x	x	
Physical examination***	x	x	x
Subjective complaints or symptoms	x	x	x
Adverse events		x	x
Resolution/follow up of adverse event			x (if applicable)
HIV related or CDC-defined AIDS defining illness	x	x	x
Resolution/follow up of HIV or AIDS illness		x	x (if applicable)
Height	x	x	
Weight	x	x	x
Medications taken†	x	x	x
Serum chemistry††	x	x	x
Serum micronutrient levels†††		x	x
Hematology‡	x	x	x
Coagulation tests (INR)	x	x	x
Plasma HIV RNA	x	x	x
Lymphocyte measures‡‡	x	x	x
HIV serology#	x		
Hepatitis serology¶		x	
Quality of life questionnaires‖		x	x
Residual pill count			x
HATS Adherence questionnaire			x
Dispense study medications		x	x (up to Week 84)
Urinalysis (microscopy only if indicated)	x	x	x
Pregnancy test§	x	x	

Test results are acceptable if results of testing within one month of visit are available
*Follow up quarterly visits may be scheduled within six days of visit date. Study termination visit will be 2-6 weeks after the Week 96 visit or premature withdrawal from the study
**Medical history including previously diagnosed medical conditions, previous opportunistic infections, and previous and current medications, including ART, alcohol, illicit and recreational drug, and natural health product use
***Complete physical examination at screening, end of study (Week 96 or premature withdrawal); targeted physical exam at other visits
†Medications including ART, alcohol, illicit and recreational drug, and natural health product use
††Glucose, blood urea nitrogen (BUN), creatinine, total protein, C-reactive protein, albumin, alkaline phosphatase, alanine transaminase (ALT), aspartate transaminase (AST), total bilirubin
†††Serum carotene (quarterly), vitamin B_{12} (quarterly), folate (six monthly), and vitamin D (25-OHD, six monthly)
‡White blood cell (WBC) count with differential, red blood cell count (RBC), hemoglobin, hematocrit, MCV, ESR, platelet count
‡‡Absolute lymphocyte count (ALC), CD4+, CD3+, and CD8+ cell counts, CD4%, CD8%, CD4:CD8 ratio. Each participant will have two measures of CD4+ cells at least one week apart to confirm each of baseline (and up to six weeks apart), outcome (<350 cells/mm^3) and end of study (96 weeks or premature withdrawal) values
#Test, if result not previously documented
¶(HBV DNA or HBsAg) and (HCV RNA or HCV Ab); test, if result not previously documented
‖MOS-HIV, EuroQol, and Health Utilities Index (HUI)
§If urine pregnancy test is positive, a serum β-HCG test will be performed. After the screening and baseline visits, a pregnancy test will be done only as needed (e.g., if menses are delayed)

REFERENCES

1. Bogden JD, Baker H, Frank O, et al. Micronutrient status and human immunodeficiency virus (HIV) infection. *Ann N Y Acad Sci* 1990;**587:**189-95.
2. Beach RS, Mantero-Atienza E, Shor-Posner G, et al. Specific nutrient abnormalities in asymptomatic HIV-1 infection. *Aids* 1992;**6**(7)**:**701-8.
3. Lacey CJ, Murphy ME, Sanderson MJ, Monteiro EF, Vail A, Schorah CJ. Antioxidant-micronutrients and HIV infection. *Int J STD AIDS* 1996;**7**(7)**:**485-9.
4. Skurnick JH, Bogden JD, Baker H, et al. Micronutrient profiles in HIV-1-infected heterosexual adults. *J Acquir Immune Defic Syndr Hum Retrovirol* 1996;**12**(1)**:**75-83.
5. Baum M, Cassetti L, Bonvehi P, Shor-Posner G, Lu Y, Sauberlich H. Inadequate dietary intake and altered nutrition status in early HIV-1 infection. *Nutrition* 1994;**10**(1)**:**16-20.
6. Baum MK, Shor-Posner G, Zhang G, et al. HIV-1 infection in women is associated with severe nutritional deficiencies. *J Acquir Immune Defic Syndr Hum Retrovirol* 1997;**16**(4)**:**272-8.
7. Semba RD, Graham NM, Caiaffa WT, Margolick JB, Clement L, Vlahov D. Increased mortality associated with vitamin A deficiency during human immunodeficiency virus type 1 infection. *Arch Intern Med* 1993;**153**(18)**:**2149-54.
8. Baum MK, Shor-Posner G, Lu Y, et al. Micronutrients and HIV-1 disease progression. *Aids* 1995;**9**(9)**:**1051-6.
9. Stephensen CB. Vitamin A, beta-carotene, and mother-to-child transmission of HIV. *Nutr Rev* 2003;**61**(8)**:**280-4.
10. Semba RD. The role of vitamin A and related retinoids in immune function. *Nutr Rev* 1998;**56**(1 Pt 2)**:**S38-48.
11. Moriguchi S, Muraga M. Vitamin E and immunity. *Vitam Horm* 2000;**59:**305-36.
12. Kupka R, Fawzi W. Zinc nutrition and HIV infection. *Nutr Rev* 2002;**60**(3)**:**69-79.
13. Keating J, Bjarnason I, Somasundaram S, et al. Intestinal absorptive capacity, intestinal permeability and jejunal histology in HIV and their relation to diarrhoea. *Gut* 1995;**37**(5)**:**623-9.
14. Sappey C, Leclercq P, Coudray C, Faure P, Micoud M, Favier A. Vitamin, trace element and peroxide status in HIV seropositive patients: asymptomatic patients present a severe beta-carotene deficiency. *Clin Chim Acta* 1994;**230**(1)**:**35-42.
15. Allard JP, Aghdassi E, Chau J, Salit I, Walmsley S. Oxidative stress and plasma antioxidant micronutrients in humans with HIV infection. *Am J Clin Nutr* 1998;**67**(1)**:**143-7.
16. Schwarz KB. Oxidative stress during viral infection: a review. *Free Radic Biol Med* 1996;**21**(5)**:**641-9.
17. Treitinger A, Spada C, Verdi JC, et al. Decreased antioxidant defence in individuals infected by the human immunodeficiency virus. *Eur J Clin Invest* 2000;**30**(5)**:**454-9.
18. Irlam JH, Visser ME, Rollins N, Siegfried N. Micronutrient supplementation in children and adults with HIV infection. *Cochrane Database Syst Rev* 2005(4)**:**CD003650.
19. Tang AM, Lanzillotti J, Hendricks K, et al. Micronutrients: current issues for HIV care providers. *Aids* 2005;**19**(9)**:**847-61.
20. Singhal N, Austin J. A clinical review of micronutrients in HIV infection. *J Int Assoc Physicians AIDS Care (Chic Ill)* 2002;**1**(2)**:**63-75.
21. Kaiser JD, Campa AM, Ondercin JP, Leoung GS, Pless RF, Baum MK. Micronutrient supplementation increases CD4 count in HIV-infected individuals on highly active antiretroviral therapy: a prospective, double-blinded, placebo-controlled trial. *J Acquir Immune Defic Syndr* 2006;**42**(5)**:**523-8.

22. Connick E, Lederman MM, Kotzin BL, et al. Immune reconstitution in the first year of potent antiretroviral therapy and its relationship to virologic response. *J Infect Dis* 2000;**181**(1)**:**358-63.
23. Koletar SL, Williams PL, Wu J, et al. Long-term follow-up of HIV-infected individuals who have significant increases in CD4+ cell counts during antiretroviral therapy. *Clin Infect Dis* 2004;**39**(10)**:**1500-6.
24. Cifone MG, Alesse E, Di Marzio L, et al. Effect of L-carnitine treatment in vivo on apoptosis and ceramide generation in peripheral blood lymphocytes from AIDS patients. *Proc Assoc Am Physicians* 1997;**109**(2)**:**146-53.
25. Moretti S, Famularo G, Marcellini S, et al. L-carnitine reduces lymphocyte apoptosis and oxidant stress in HIV-1-infected subjects treated with zidovudine and didanosine. *Antioxid Redox Signal* 2002;**4**(3)**:**391-403.
26. Breitkreutz R, Pittack N, Nebe CT, et al. Improvement of immune functions in HIV infection by sulfur supplementation: two randomized trials. *J Mol Med* 2000;**78**(1)**:**55-62.
27. Droge W, Breitkreutz R. N-acetyl-cysteine in the therapy of HIV-positive patients. *Curr Opin Clin Nutr Metab Care* 1999;**2**(6)**:**493-8.
28. Cavallini L, Alexandre A. Oral N-acetyl-cysteome increases the production of anti HIV chemokines in peripheral blood mononuclear cells. *Life Sci* 2000;**67**(2)**:**147-54.
29. Cossarizza A, Mussini C, Mongiardo N, et al. Mitochondria alterations and dramatic tendency to undergo apoptosis in peripheral blood lymphocytes during acute HIV syndrome. *Aids* 1997;**11**(1)**:**19-26.
30. Cossarizza A, Franceschi C, Monti D, et al. Protective effect of N-acetylcysteine in tumor necrosis factor-alpha-induced apoptosis in U937 cells: the role of mitochondria. *Exp Cell Res* 1995;**220**(1)**:**232-40.
31. Hart AM, Wilson AD, Montovani C, et al. Acetyl-l-carnitine: a pathogenesis based treatment for HIV-associated antiretroviral toxic neuropathy. *Aids* 2004;**18**(11)**:**1549-60.
32. Baur A, Harrer T, Peukert M, Jahn G, Kalden JR, Fleckenstein B. Alpha-lipoic acid is an effective inhibitor of human immuno-deficiency virus (HIV-1) replication. *Klin Wochenschr* 1991;**69**(15)**:**722-4.
33. Jiamton S, Pepin J, Suttent R, et al. A randomized trial of the impact of multiple micronutrient supplementation on mortality among HIV-infected individuals living in Bangkok. *Aids* 2003;**17**(17)**:**2461-2469.
34. Fawzi WW, Msamanga GI, Spiegelman D, et al. Randomised trial of effects of vitamin supplements on pregnancy outcomes and T cell counts in HIV-1-infected women in Tanzania. *Lancet* 1998;**351**(9114)**:**1477-82.
35. Fawzi WW, Msamanga GI, Spiegelman D, et al. A randomized trial of multivitamin supplements and HIV disease progression and mortality. *N Engl J Med* 2004;**351**(1)**:**23-32.
36. Alexander M, Newmark H, Miller RG. Oral beta-carotene can increase the number of OKT4+ cells in human blood. *Immunol Lett* 1985;**9**(4)**:**221-4.
37. Baeten JM, McClelland RS, Wener MH, et al. Relationship between markers of HIV-1 disease progression and serum beta-carotene concentrations in Kenyan women. *Int J STD AIDS* 2007;**18**(3)**:**202-6.
38. Austin J, Singhal N, Voigt R, et al. A community randomized controlled clinical trial of mixed carotenoids and micronutrient supplementation of patients with acquired immunodeficiency syndrome. *Eur J Clin Nutr* 2006;**60**(11)**:**1266-76.

39. Baum MK, Mantero-Atienza E, Shor-Posner G, et al. Association of vitamin B6 status with parameters of immune function in early HIV-1 infection. *J Acquir Immune Defic Syndr* 1991;**4**(11)**:**1122-32.
40. Rall LC, Meydani SN. Vitamin B6 and immune competence. *Nutr Rev* 1993;**51**(8)**:**217-25.
41. Hepburn MJ, Dyal K, Runser LA, Barfield RL, Hepburn LM, Fraser SL. Low serum vitamin B12 levels in an outpatient HIV-infected population. *Int J STD AIDS* 2004;**15**(2)**:**127-33.
42. Tang AM, Graham NM, Chandra RK, Saah AJ. Low serum vitamin B-12 concentrations are associated with faster human immunodeficiency virus type 1 (HIV-1) disease progression. *J Nutr* 1997;**127**(2)**:**345-51.
43. Breitkreutz R, Holm S, Pittack N, et al. Massive loss of sulfur in HIV infection. *AIDS Res Hum Retroviruses* 2000;**16**(3)**:**203-9.
44. Rahman MM, Vermund SH, Wahed MA, Fuchs GJ, Baqui AH, Alvarez JO. Simultaneous zinc and vitamin A supplementation in Bangladeshi children: randomised double blind controlled trial. *Bmj* 2001;**323**(7308)**:**314-8.
45. Karyadi E, West CE, Schultink W, et al. A double-blind, placebo-controlled study of vitamin A and zinc supplementation in persons with tuberculosis in Indonesia: effects on clinical response and nutritional status. *Am J Clin Nutr* 2002;**75**(4)**:**720-7.
46. CDC. 1993 revised classification system for HIV infection and expanded surveillance case definition for AIDS among adolescents and adults. 1992. *MMWR Recomm Rep*;**41**(RR-17)**:**1-19.
47. Cook NR, Albert CM, Gaziano JM, et al. A randomized factorial trial of vitamins C and E and beta carotene in the secondary prevention of cardiovascular events in women: results from the Women's Antioxidant Cardiovascular Study. *Arch Intern Med* 2007;**167**(15)**:**1610-8.
48. Bjelakovic G, Nikolova D, Gluud LL, Simonetti RG, Gluud C. Mortality in randomized trials of antioxidant supplements for primary and secondary prevention: systematic review and meta-analysis. *Jama* 2007;**297**(8)**:**842-57.
49. The effect of vitamin E and beta carotene on the incidence of lung cancer and other cancers in male smokers. The Alpha-Tocopherol, Beta Carotene Cancer Prevention Study Group. *N Engl J Med* 1994;**330**(15)**:**1029-35.
50. Tang AM, Graham NM, Saah AJ. Effects of micronutrient intake on survival in human immunodeficiency virus type 1 infection. *Am J Epidemiol* 1996;**143**(12)**:**1244-56.
51. McComsey G, Southwell H, Gripshover B, Salata R, Valdez H. Effect of antioxidants on glucose metabolism and plasma lipids in HIV-infected subjects with lipoatrophy. *J Acquir Immune Defic Syndr* 2003;**33**(5)**:**605-7.
52. Doherty CP. Host-pathogen interactions: the role of iron. *J Nutr* 2007;**137**(5)**:**1341-4.
53. Semba RD, Ricketts EP, Mehta S, et al. Effect of Micronutrients and Iron Supplementation on Hemoglobin, Iron Status, and Plasma Hepatitis C and HIV RNA Levels in Female Injection Drug Users: A Controlled Clinical Trial. *J Acquir Immune Defic Syndr* 2007;**45**(3)**:**298-303.
54. Hammer SM, Eron JJ, Jr., Reiss P, et al. Antiretroviral treatment of adult HIV infection: 2008 recommendations of the International AIDS Society-USA panel. *Jama* 2008;**300**(5)**:**555-70.
55. Balfour L, Tasca GA, Kowal J, et al. Development and validation of the HIV Medication Readiness Scale. *Assessment* 2007;**14**(4)**:**408-16.

56. HIV Diagnosis at CD4 Count Above 500 Cells/mm3 and Progression to Below 350 Cells/mm3 Without Antiretroviral Therapy. *J Acquir Immune Defic Syndr* 2007;**46**(3)**:**257-8.
57. Clinical outcomes from OPTIMA: A randomized controlled trial of antiretroviral treatment interruption of intensification interruption or intensification in advance multi-drug resistant HIV infection. OPTIMA Team including DW Cameron. Abstract. *International Aids Conference. Mexico City.* August 3-8, 2008.

APPENDIX A

List of Events and Conditions Considered Manifestations of HIV Infection

1. Candidiasis:
   - *Bronchi
   - *Esophagus
   - *Lungs
   - Oropharyngeal (Thrush)
   - *Trachea
   - Vulvovaginal (persistent, frequent, or poorly responsive to therapy)
   - Other Candidiasis
2. Cytomegalovirus:
   - *Retinitis (with loss of vision)
   - *Cytomegalovirus Disease (other than liver, spleen or nodes)
3. Herpes Simplex Virus
   - *Bronchitis
   - *Esophagitis
   - *Pneumonitis
   - *Chronic Ulcer(s) (>1 month in duration)
   - Other Sites
4. Lymphoma (HIV-Related)
   - *Burkitt's (or equivalent term)
   - *Immunoblastic (or equivalent term)
   - *Primary of brain
5. Mycobacterial Disease:
   - **Mycobacterium avium* complex
   - **Mycobacterium kansasii,* disseminated or extra pulmonary
   - **Mycobacterium tuberculosis,* any site (pulmonary or extrapulmonary)
   - *Other *Mycobacterial* species, disseminated or extrapulmonary
6. Other
   - *Cervical cancer, invasive
   - Cervical cancer, *in situ*
   - Cervical dysplasia
   - *Coccidioidomycosis, disseminated or extra pulmonary
   - *Cryptococcis, extra pulmonary
   - *Cryptosporidiosis, chronic intestinal (>1 month's duration)
   - *Encephalopathy HIV-related
   - Hairy leukoplakia, oral
   - Herpes zoster
   - *Histoplasmosis, disseminated or extrapulmonary
   - Isosporiasis
   - Kaposi's sarcoma
   - Listeriosis
   - **Pneumocystis carinii* pneumonia
   - *Pneumonia, recurrent
   - *Progressive multifocalleukoencephalopathy
   - Salmonella septicemia, recurrent
   - *Toxoplasmosis of the brain
   - *Wasting syndrome due to HIV

*AIDS-defining event as described by CDC Surveillance Case Definition of 1993

APPENDIX B

Details of Sample Size Calculation

Two-Sample Log-Rank Power Analysis

Event: CD4 count <350 cells/mm^3 or emergence of documented CDC-defined AIDS-defining illness or start of ART
Null Hypothesis: Median K-PAX Event Rate at 2 years = Median Control Event Rate at 2 years
Alternative Hypothesis: Median K-PAX Event Rate at 2 years <> Median Control Event Rate at 2 years

Power	Sample Size	Event rate K-PAX	Event rate Control	Accrual Time	Follow-up Time	Non-Compliance K-PAX	Non-compliance Control
80%	101	50%	24%	1	2	15%	20%
80%	120	50%	26%	1	2	15%	20%
80%	143	50%	28%	1	2	15%	20%
80%	175	50%	30%	1	2	15%	20%
**80%**	**218**	**50%**	**32%**	**1**	**2**	15%	20%
80%	276	50%	34%	1	2	15%	20%
80%	363	50%	36%	1	2	15%	20%
80%	496	50%	38%	1	2	15%	20%

Base Time: 2 years
Proportion loss to follow up in K-PAX during the base time: 0.15000
Proportion loss to follow up in control during the base time: 0.20000
Percent of accrual time until 50% enrollment is reached: 50.00%

Report Definitions
Power is the probability of rejecting a false null hypothesis. Power should be close to one.
Alpha is the probability of rejecting a true null hypothesis. It should be small.
Beta is the probability of accepting a false null hypothesis. It should be small.

Summary
A two-sided log rank test with an overall sample size of 218 subjects (of which 109 are in control group 1 and 109 are in the treatment group) achieves 80% power at a 0.05 significance level to detect a difference of 18% between the proportions achieving the outcome in the control and treatment groups after 2 years. Participants will be enrolled during an accrual period of 1 year and 50% of the enrollment will be complete when 50% of the accrual time has past. We have also accounted for a modest non-compliance rate of approximately 15% over the two years for those participants allocated to active treatment medication and 20% for those allocated to control.

Chart

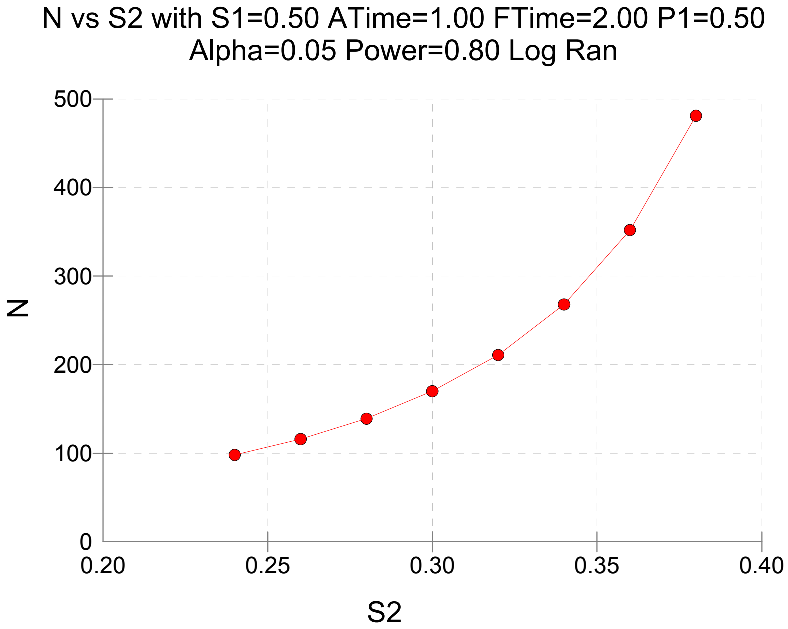
N vs S2 with S1=0.50 ATime=1.00 FTime=2.00 P1=0.50
Alpha=0.05 Power=0.80 Log Ran
N
500
400
300
200
100
0
0.20
0.25
0.30
0.35
0.40
S2

APPENDIX C

Composition of Medications (Manufacturer: K-PAX (USA)

**High Potency Antioxidant Formula (K-PAX Ultra®)**			**Low Dose Multivitamins** (equivalent to Centrum®)		
	**Daily Dosage***	**Percent RDA†**		**Daily Dosage**	**Percent RDA**
**Vitamins**			**Vitamins**		
Vitamin A (100% b carotene)	20,000 IU	400	Vitamin A (29% b carotene)	3,500 IU	70
Vitamin C	2,000 mg	3000	Vitamin C	60 mg	100
Vitamin E (a tocopherol)	200 IU	667	Vitamin D3	400 IU	100
Vitamin B-1	60 mg	4000	Vitamin E (a tocopherol)	30 IU	100
Vitamin B-2	60 mg	3530	Vitamin B-1	1.5 mg	100
Vitamin B-6	200 mg	10000	Vitamin B-2	1.7 mg	100
Niacinamide	60 mg	300	Vitamin B-6	2 mg	100
Pantothenic Acid	60 mg	600	Niacin	20 mg	100
Folic Acid (Folacin)	1 mg	250	Pantothenic Acid	10 mg	100
Biotin	600 mcg	200	Folic Acid	400 mcg	100
Vitamin D3	400 IU	100	Vitamin B12	6 mcg	100
Vitamin B12	2.5 mg	41666			
Bioflavinoid Complex	300 mg	-			
Mixed tocopherols	200 IU	-			
Choline (Citrate)	60 mg	-			
Inositol	60 mg	-			
**Minerals**			**Minerals**		
Calcium	800 mg	80	Calcium	162 mg	16
Magnesium (Citrate)	400 mg	100	Magnesium	100 mg	25
Iron (Picolinate)	18 mg	100	Iron	18 mg	100
Iodine (K iodide)	150 mcg	100	Phosphorous	109 mg	11
Copper (Picolinate)	2 mg	100	Iodine	150 mcg	150
Manganese(Picolinate)	10 mg	500	Copper	2 mg	100
Potassium (Citrate)	99 mg	2	Manganese	2 mg	100
Zinc (Picolinate)	30 mg	200	Potassium	80 mg	2
Selenium (Picolinate)	200 mcg	286	Zinc	15 mg	100
Chromium (Picolinate)	100 mcg	84	Selenium	20 mcg	29
Molybdenum (Picolinate)	300 mcg	400	Chromium	120 mcg	100
Boron (Picolinate)	2 mg	-	Molybdenum	75 mcg	75
Betaine HCL	150 mg	-			
Glutamic Acid HCL	400 mg	-			
**Potent Antioxidants**					
Alpha Lipoic Acid	400 mg	-			
Acetyl L-Carnitine	1000 mg	-			
N-Acetyl Cysteine	1200 mg	-			

*Divided in 16 capsules, taken as one packet of 8 capsules twice daily
†Recommended daily allowance
